# Electrostatic Spray Disinfection Using Nano-Engineered Solution on Frequently Touched Surfaces in Indoor and Outdoor Environments

**DOI:** 10.3390/ijerph19127241

**Published:** 2022-06-13

**Authors:** Tanya Purwar, Shamya Dey, Osama Zaid Ali Al-Kayyali, Aaron Floyd Zalar, Ali Doosttalab, Luciano Castillo, Victor M. Castano

**Affiliations:** 1 School of Mechanical Engineering, Purdue University, West Lafayette, IN 47907, USA; shamyadey@gmail.com (S.D.); osamalkali@gmail.com (O.Z.A.A.-K.); aazalar@gmail.com (A.F.Z.); ali.doosttalab@gmail.com (A.D.); castil63@purdue.edu (L.C.); 2 Centro de Física Aplicada Tecnología Avanzada, Universidad Nacional Autónoma de México, Juriquilla, Querétaro 76230, Mexico; meneses@unam.mx

**Keywords:** COVID-19, pathogens, disinfection, nano-disinfectant, electrostatic spray deposition, fomites

## Abstract

The COVID-19 pandemic has resulted in high demand for disinfection technologies. However, the corresponding spray technologies are still not completely optimized for disinfection purposes. There are important problems, like the irregular coverage and dripping of disinfectant solutions on hard and vertical surfaces. In this study, we highlight two major points. Firstly, we discuss the effectiveness of the electrostatic spray deposition (ESD) of nanoparticle-based disinfectant solutions for systematic and long-lasting disinfection. Secondly, we show that, based on the type of material of the substrate, the effectiveness of ESD varies. Accordingly, 12 frequently touched surface materials were sprayed using a range of electrostatic spray system parameters, including ion generator voltage, nozzle spray size and distance of spray. It was observed that for most cases, the surfaces become completely covered with the nanoparticles within 10 s. Acrylic, Teflon, PVC, and polypropylene surfaces show a distinct effect of ESD and non-ESD sprays. The nanoparticles form a uniform layer with better surface coverage in case of electrostatic deposition. Quantitative variations and correlations show that 1.5 feet of working distance, an 80 μm spray nozzle diameter and an ion generator voltage of 3–7 kV ensures a DEF (differential electric field) that corresponds to an optimized charge-to-mass ratio, ensuring efficient coverage of nanoparticles.

## 1. Introduction

The United States Centers for Disease Control and Prevention (US-CDC) has published that, annually, up to 650,000 deaths are associated with respiratory diseases [1]. Respiratory viruses, novel or mutated, have been a cause of medical conundrums for centuries now. In recent decades, the world has seen epidemics and pandemics caused by respiratory infections such as severe acute respiratory syndrome coronavirus (SARS-CoV-1), Middle East respiratory syndrome (MERS) coronavirus, and influenza virus [2]. In December 2019, the first case of novel severe acute respiratory syndrome coronavirus 2 (SARS-CoV-2) disease was reported. Commonly addressed as COVID-19 infection, it was soon declared a pandemic [3]. Many researchers have attempted to quantify the pathways that lead to the transmission of this deadly virus so as to curb its rapid spread. There are different routes of inter-human virus transmission. Direct human-to-human contact or via intermediate fomites, aerosols or droplets generated during coughing, sneezing or talking are the multiple means of this vector spread [4,5]. These microdroplets might remain suspended in air or settle on inanimate environmental surfaces. Although direct contact is the predominant route of virus transmission, it is difficult to quantify indirect routes of spread through fomites. This leads to difficulty in contact tracing [6] that could facilitate rapid and unaccounted dissemination of the respiratory virus [7]. Humans tend to touch their eyes, nose and mouth up to about 200 times a day [8]. When one touches a contaminated surface and then touches their face, they introduce the pathogens into their body. Rusin et al. demonstrated that 34% of viruses can be transmitted to the mouth and 64% can be transferred to uncontaminated hands [9].

Studies have been conducted to determine the role of inanimate objects in the vicinity of patients or in high viral load environments [10]. This is to quantify the role of surfaces in transmission of such pathogens [11]. Doremalen et al. [12] applied the SARS-CoV-2 virus on plastic, stainless steel, copper and cardboard. The viable virus was detected in stable condition after 72 h of application on these surfaces. In another study [13], multiple swabs were taken from COVID positive patients’ room and from personal protective equipment (PPE) of staff. These swabs were tested for viral presence, and results were positive. It was observed that after routine cleaning of the spaces and the PPE, the samples collected with the swabs were negative. In the Pancic study [14], 3 to 1800 PFU of rhinovirus was recovered from the fingertips of volunteers who touched contaminated doorknobs and faucets. These studies suggest that protective measures and regular decontamination of inanimate surfaces can reduce the risk of fomite transmission in different environmental settings. During the pandemic, many professional and recreational sites, such as transportation, educational institutions, banks and offices, shopping centers, and so on, have suffered from close-downs and losses because of the lack of knowledge about effective disinfection against such a rapidly spreading disease. In order to curb virus spread through the various means of transmission, there has been a rise in awareness about social distancing [15], wearing masks [16], washing hands [17] and disinfecting our surroundings. To fight transmission through fomites, it is imperative that more understanding of disinfection procedures and availability of correct resources is acquired so as to return to a normal course of livelihood [18,19].

In an indoor surrounding, one can be in contact with different types of surface materials that can be categorized as porous or non-porous surfaces. Hard surfaces and vertical surfaces are particularly difficult to clean due to dripping of the disinfectant solution. The uncertainty about proper coverage through the surface, amount of applied solution, required time for the solution in inactivating the pathogens, effective longevity of disinfectants, etc., are some of the many open questions that need quantified explanations to improve the current disinfection standards. As much as the type of disinfectant solution and its properties play a role in the process, understanding the type of material being decontaminated is also important. Rakowska et al. [20] points four important properties that can affect the persistence of viruses on surfaces [21]. These include (i) physical surface properties such as porosity and factors like the type of surface, e.g., roughness; (ii) environmental factors such as temperature, relative humidity and exposure to light; (iii) virus structure; and (iv) chemical conditions such as pH, adsorption state or presence of a specific element or organisms. Huslage et al. [11] categorized different objects in a healthcare setting as “high touch”, “medium touch” and “low touch” based on how frequently these surfaces come in direct contact with healthcare workers and patients, thus helping in quantifying the frequency of disinfection required. Kampf et al. [22] tabulated the ideal temperature and time of persistence of human coronavirus on inanimate surfaces like glass, copper, polypropylene, stainless steel, etc., and concluded that human coronavirus can remain infectious on such inanimate surfaces for up to 9 days at room temperature. They studied the effect of biocidal agents for virus inactivation, and quantified the transmissibility of influenza virus from contaminated surface to hands. Human coronavirus can persist on hard non-porous surfaces for long hours and even weeks in some cases [20]. Lai et al. [23] highlighted that absorbent materials can offer more protection against virus containing respiratory droplets. Chattopadhyay et al. [24] highlighted that the absorption of viruses with a hydrophobic outer layer was favored by surfaces coated by hydrophobic sorbents, while hydrophilic viruses were absorbed by hydrophilic surfaces. Such studies highlight the urgent need for accounting for common surface materials when designing the methodologies of disinfection for particular spaces.

During this global emergency, there has been an increase in the use of electrostatic spray technology for disinfection [25,26]. Several agencies have undertaken this method for safe and reliable disinfection. In the electrostatic spray deposition (ESD) technique [27], a potential difference is applied over the disinfectant solution exiting the nozzle. This builds electric charge near the surface of the nascent droplets at the exit, which compensates for the surface tension. As a result, the droplet surface becomes unstable and spontaneously breaks up into smaller and mono-dispersed charged droplets [28,29]. The charged droplets do not coalesce in air since they all have the same charge (like charges repel) and follow a trajectory to the nearest grounded surface, which is the target substrate. Since charged droplets are electrostatically attracted to the grounded object, this method exhibits a higher transfer efficiency compared to uncharged spraying methods [30,31,32]. The electrostatic spray system has been extensively used in the paint and coating industry as well as the agriculture industry [33]. Several models [34,35] have been developed to understand the deposition behavior of electrostatic sprays, as well as their particle emission rates and properties. These highlight the effectiveness of using an electrostatic spray technique with particle-based precursor solutions. In recent times, nanotechnology-based solutions composed of nano-particles are becoming popular [36], and the conflation of ESD and nanoparticles holds a great potential for disinfection applications.

Chintagunta et al. [37] reports that under different operation conditions, alcohol- and water-based disinfectants may not work at their best efficiency, resulting in the need for better and persistent disinfection solutions. Nanotechnology opens a new avenue for developing efficient disinfectant systems with antimicrobial activity and self-cleaning ability. Significant research has been conducted on the study and application of some specific types of nanoparticles, given that their inherent anti-microbial and, in some cases, anti-viral activity prevents the pathogens from generating resistance against the disinfectant [38]. This is unlike many anti-microbial solutions that rely exclusively on the chemical disruption of the microorganisms. Such chemicals can also cause disinfectant-induced hormesis on non-target organisms [39]. Studies have confirmed that some metallic and metal oxide nanoparticles are of particular interest due to their inherent anti-microbial properties that could also be enhanced by synergistic combinations of particular compositions and structures [40]. Singh et al. [41] suggests that silver nanoparticles have potent antimicrobial properties and are one of the most useful metal disinfectants against viruses, bacteria and other pathogens. Cavalcanti et al. [42] talks about the use of silver nanoparticles in disinfectants that can be used in handwashing, which ensures that it kills 99% of germs and bacteria. They also discuss the use of a nano-disinfectant for cleaning door handles, elevator buttons, and a rear telephone, protecting them against the SARS-CoV-2 virus. Campos et al. [36] presents a table that summarizes manuscripts found in the literature and patents related to disinfectants and sanitizers based on nanotechnology. More studies such as [43,44,45,46,47] show that nanoparticles can be integrated into solutions to confer sterilizing properties. Such nano-products play a significant role in optimized cleaning and the long-term persistence of active disinfection and inactivation of microbes on contaminated surfaces.

Along with spraying nano-antimicrobial and antiviral solutions, other nanotechnology-based strategies have also been recognized to curb virus spread. These include antiviral surface coatings that can potentially kill viruses that persist on various surface materials [48,49,50,51,52]. Homaeigohar et al. [53] reviewed how antiviral nanohybrids could potentially be applied to surfaces for virus inactivation. They discussed the use of Ag (silver) nanoparticles and ZnO (zinc oxide) nanorods for promising antiviral performance against influenza virus and coronavirus, antibacterial activity for Gram-negative and -positive bacteria, and photocatalytic decomposition of organic pollutants [54]. The promising antiviral activity of carbon nanomaterials is also discussed. Pemmada et al. [55] talked about the use of antiviral hybrid nanomaterials to achieve viricidal effects. Nanomaterials have the property to follow viruses into host cells, destroy cellular and viral factors and block the replication of virus particles. Looking at the potential effectiveness of nanotechnology against the emerging pandemic as well as the efficiency of electrostatic spray deposition for disinfection, the amalgamation of two can be of great advantage against the spread of respiratory viruses through fomites.

The combination of electrostatic spraying and nano-disinfectants with appropriate system parameters, such as the charge density, flow rate and working distance (distance of spray nozzle to target surface), can help in the uniform deposition of anti-viral nanoparticles, leading to an effective disinfection process [56]. The standard default system settings of existing foggers and sprayers that are currently used and commercially available might not suit all materials and environmental conditions. For example, the sprayer settings that are suitable for surfaces inside an aircraft cabin might not be suitable for a classroom. It is an indispensable need of the hour to establish reliable strategies for disinfection so that we can fight the current pandemic as well as continue safe practices post-pandemic [57]. The purpose of this study is to establish an optimized system of parameters (voltage, nozzle size/flow rate, working distance) that can help in improving the existing process of electrostatic disinfection of porous and non-porous surfaces using nano-engineered precursor solution in various environmental settings involving medical facilities, airports, universities and other public spaces.

## 2. Materials and Methods

In this study, we used parts from the VP200ES Professional Cordless Electrostatic Handheld Sprayer in collaboration with Victory Innovations to build a prototype electrostatic sprayer with a changeable ion generator for variable voltages and a gear key system for automatically changing the nozzle size through a remote control. The prototype was used to spray a nano-disinfectant on 10 common surfaces that were identified as frequently touched and contaminated in healthcare settings and other public spaces. The samples were observed under a scanning electron microscope (SEM) to compare between charged and uncharged spraying of nanoparticle-based disinfectant precursor solution. The samples were tested for varying electrostatic spray system parameters, and observations were noted.

### 2.1. Frequently Touched Surfaces

The global infection control community has stated that contaminated environmental surfaces are an important contributing factor to the transmission of pathogens [58]. Different kinds of viruses use varying mechanisms and chemistry to attach and enter a host cell. In most cases, viral entry occurs when a viral spike protein attaches to a receptor on a host cell surface, followed by low pH fusion and replication [59]. Respiratory viruses, including human coronavirus, can remain active and infectious on inanimate surfaces at room temperature for several days [60]. These substrate materials are a potential route of virus transmission, either by touch from an infected person or if someone coughs or sneezes around them. Studies [61,62,63] show quantitative observations on how frequently some objects are touched when in contact with patients and medical providers in healthcare settings. Such objects are highly contaminated. Some studies [64,65] have highlighted the persistence of human coronavirus on various metals, polymers and wood. The inherent properties of these materials is very crucial in the stable or unstable survival of viruses on them. Chaturvedi et al. [66] highlights that metal ions play an important role in the survival and pathogenesis of a large group of viruses. Metal binding on a structural basis can be useful in the early design and development of viral inhibitors. The virucidal and biocidal properties of metals like copper and silver are well documented [20,67,68,69,70]. Mouritz et al. [71] discusses how polymer matrix composite materials can aid the attachment of respiratory viruses to the polymer substrata and retain it when in contact with infected people sneezing or coughing in close proximity. They show that SARS-CoV-1 has been found to be retained on plastic surfaces for 3–4 days, although some existing data suggest that viral persistence on plastic can be as high as 28 days. Such varying survival time (1–28 days) in the case of plastics is because it is dependent on multiple factors like virus initial concentration, surface conditions (roughness, charge, and wettability), humidity, and temperature. These surface materials are present around us in common public places like classrooms, offices, airports, hospitals, restaurants, gym and in almost any indoor setting. In this study, we selected 12 surfaces that represent different levels of surface physical properties, such as porosity, absorption and surface hydrophobicity. The test surfaces include: *(i) copper; (ii) aluminum; (iii) cast iron; (iv) stainless steel; (v) polyethylene plastic; (vi) PVC; (vii) polypropylene; (viii) Teflon; (ix) rubber; (x) aircraft 1; (xi) aircraft 2; and (xii) wood*. The aircraft 1 sample is Schneller Aerfilm decorative laminate on a phenolic composite stackup, and the aircraft 2 sample is polyurethane Mankiewicz Bioprotect Topcoat on Ultem 9085. Aircraft 1 and 2 are the materials used in an airplane interior cabin. These were obtained for this test in collaboration with Raytheon Technologies.

### 2.2. Nanoparticle-Based Disinfectant

**Figure 1:**
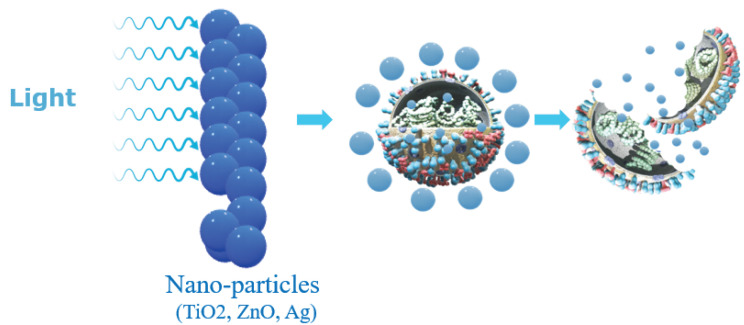
Action of Nanoxen on pathogens (Taken from Nanoxen-Nano Coatings Technologies).

The nanoparticle-based disinfectant solution used in this study is called Nanoxen (http://www.nanoxenmexico.com/, accessed on 2 June 2022), which is a novel nanotechnology-based solution. Nanoxen is made of various inorganic nanoparticles, including titania, silica, zinc oxide, silver and specially made core-shell nanostructures. They have been chemically functionalized to be effectively dispersed into a water-borne suspension. It claims disinfection with both microbicidal and microbiostatic properties by using mono- and multi-component nanostructures selected from one or more suitable inorganic nanoparticles, one or more ceramic nanoparticles and one or more carbonaceous nanoparticles. The patented composition of Nanoxen (https://trademarks.justia.com/902/42/nanoxen-90242121.html, accessed on 2 June 2022) includes nano-particles with a photocatalytic behavior. According to the literature [72,73,74], the disinfection properties of photocatalysis at the nano scale is attributed to the generation of reactive oxygen species on the surface of the nanoparticles. Furthermore, its disinfection capacity and overall performance can be significantly improved through surface, shape and size modifications of the photocatalytic nanomaterial. Additionally, the interaction of light, specifically the entire UV through IR electromagnetic spectrum, with the nanostructures results in the formation of free metal ions. In general, the bactericidal and anti-viral activity of nanoparticles are known to depend on size, stability and concentration in the growth medium. This is because while growing in a medium modified or supplemented with nanoparticles, the microorganism’s population growth can be inhibited by some specific nanoparticle interactions. For typical respiratory viruses, their outer lipidic envelope has pores in the nano-meter range, which makes nanoparticles suitable for crossing the membrane and penetrating the virus. This produces physico-catalytical disruption of their structure instead of a purely chemical reaction, as is the case of standard disinfectants [75]. This makes compositions like Nanoxen very efficient while requiring much lower concentrations for the effective annihilation of micro- and nano-organisms while, in turn, decreasing toxicity effects [40]. Photocatalytic nanostructures have been used broadly for killing different families of micro- and nano-organisms including bacteria, fungi, lichens and viruses because they present high photo-reactivity, broad-spectrum anti-biosis and chemical stability when used on different surfaces. This allows them to decompose organic compounds by the formation and constant release of hydroxyl radicals and superoxide ions when exposed to light. These are highly efficient in inhibiting the growth of even antibiotic-resistant microorganisms, as shown in Figure 1.

More details on different nanotechnology-based disinfectants can be found in the studies cited here [37,38,43,46,76,77,78]. Inanimate surface materials are subject to wear over time. This is because of direct human contact or environmental factors. Understanding the substrate-particle adhesion for the uniform and proper deposition of anti-microbial nanoparticles is very crucial. This helps in substantiating that the anti-viral property of the solution sprayed on the surface is active for long periods of time and is effective. In the electrostatic spray deposition (ESD) process, the electrostatic image force of attraction and Coulombic force of repulsion play crucial roles in the deposition of the spray film and may control the maximum coverage over surfaces of different shapes and sizes [27,30,79,80,81]. After initial deposition on the grounded substrate, the electrostatically charged nano-particles repel new incoming particles that, in turn, tend to form a layer away from the existing charged particles, spreading around the surface. This helps in avoiding any agglomeration of nanoparticles, which is common when sprayed using a traditional spraying technique.

### 2.3. Prototype Design

**Figure 2:**
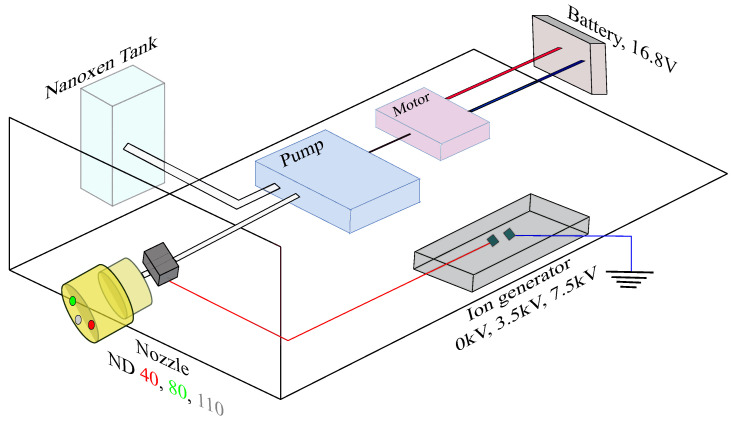
Electrostatic sprayer prototype.

A prototype electrostatic jet sprayer was assembled as shown in Figure 2. It is composed of two key functions crucial to our study. The first function is to vary the charge applied to the disinfectant prior to spraying. Parts from the Victory VP200ES handheld electrostatic sprayer were used as a base for the prototype. The charging module of the Victory handheld electrostatic sprayer works in two steps. It initially charges the charging ring at the tip of the sprayer and then works backwards to charge the contents of the tank. The disinfectant is then pumped through the nozzle, where it is charged a second time via the charge ring before it is atomized and sprayed. The built-in charging module, which is a positive ion generator, traditionally applies a potential difference of +7 kV. For the prototype, the casing of the sprayer was removed, allowing us to change the charging module. A separate positive ion generator capable of applying a potential difference of 3.5 kV was acquired. A circuit was constructed to allow us to switch between the 3.5 kV and 7 kV ion generators. This, in turn, allowed us to vary the charge applied to the spray for our experiment. The 3-in-1 nozzle of the sprayer allowed for the selection of spray particle sizes between 40 μm, 80 μm and 110 μm. This process is traditionally achieved by using a key to manually turn the nozzle to the desired setting. In our prototyping, we eliminated the manual element and introduced an automatic toggling function using a micro-controller. The stock key was redesigned so that its circumference served as a spur gear. A pinion gear driven by a NEMA 17 stepper motor was mounted on the nozzle and placed in a constant mesh with the redesigned key. An Arduino UNO was used to control the stepper motor, turning it in 120 degree increments to toggle between spray nozzle sizes.

### 2.4. Experimental Design

#### 2.4.1. Scanning Electron Microscopy Setup

A total of 20 surface samples (2 of each from Section 2.1 were taken, excluding Aircraft 1 and Aircraft 2) of 1${}^{\prime \prime }$× 1${}^{\prime \prime }$ dimensions were used for this study. From these, 10 samples were sprayed using the prototype sprayer with the voltage on at 1.5 feet working distance for 10 s. Voltage being on indicated that the bipolar ion generator was set with the default 7 kV output to charge the precursor nano-solution. The other 10 test samples were sprayed with the voltage off (0 kV ion generator output) under the same conditions. The mass flow rate was 1.5 g/s at a 40 micron nozzle size with a conical spray pattern in each case. The samples were coated with an ultra-thin coating of electrically conducting metal, silver (Ag), using the sputter coating method [82] and prepared for SEM imaging under the conditions listed in Table 1.

The samples were incubated for 24 h at room temperature, and surface scanning was done using an FEI NOVA nanoSEM Field Emission Scanning Electron Microscope at 10 kV acceleration voltage. High vacuum imaging was used for high magnification and resolution.

#### 2.4.2. Electrostatic Spray System Setup

**Figure 3:**
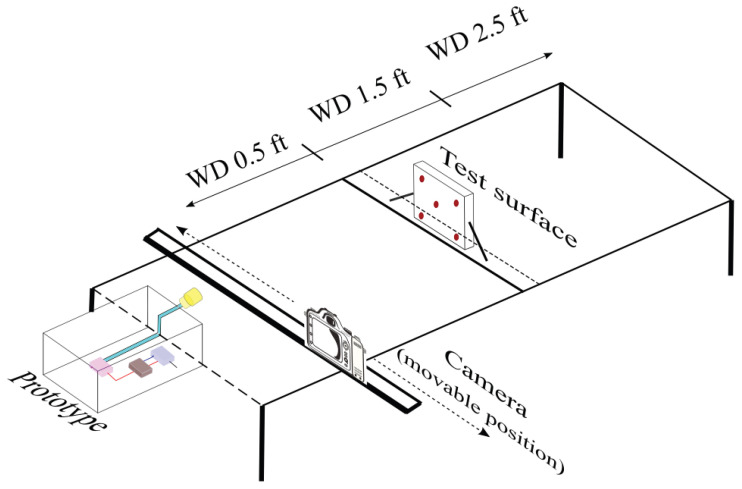
Schematic of the experimental setup of the electrostatic spray deposition for varying system parameters.

This experiment was designed to observe the effects of varying electrostatic spray system parameters on different substrate materials (Section 2.1) in a laboratory setting. The experiment includes the use of the prototype (Section 2.3) to spray the nano-disinfectant solution (Section 2.2) on 6${}^{\prime \prime }$× 6${}^{\prime \prime }$ sample surfaces fastened vertically to the test rig. The nozzle was mounted at a fixed position on one end of the test rig as shown in Figure 3. The test samples were attached to a movable beam that allowed us to change the distance between the nozzle and surface. The materials studied were wood, polyethylene, aluminum, PVC, cast iron, stainless steel, rubber, copper, polypropylene, Teflon, Aircraft 1 and Aircraft 2. The independent variables were: (i) working distance (WD), defined as the distance between the sprayer nozzle and the test substrate (0.5 ft, 1.5 ft, 2.5 ft); (ii) ion generator voltage (0 kV, 3.5 kV, 7 kV); and (iii) nozzle diameter (40 μm, 80 μm, and 110 μm). For each test, the sample surface was first positioned upright at the same horizontal level as the nozzle at its respective WD. Both qualitative and quantitative data was collected. Quantitative data were collected in terms of differential electric field (DEF), and qualitative data were collected in terms of images. Before spraying, the electric potential was measured at 5 points using a non-contact surface voltmeter (Trek 884 Non-Contacting Electrostatic Voltmeter) as shown in Figure 3. Next, the prototype was used to spray the disinfectant on the mounted target for 3 s. The electric potential on the substrate was measured again immediately after spraying at five locations as shown in Figure 3. DEF was calculated as the difference in the average voltmeter reading before and after spraying for the 5 points on the sample surface. An image of the droplet pattern was obtained using a mounted camera. The test surface was then removed, wiped and allowed to discharge over a period of time. This was repeated for every combination of the independent variables for each of the 12 test materials.

The charge-to-mass ratio is considered a crucial parameter that is used to determine the effectiveness of electrostatic spray deposition. This can be represented as the ratio of the charge (Coulomb) to the flow rate (kg/s) [81]. As per Coulomb’s law, the electric potential at a point is directly proportional to the charge and inversely proportional to the square of distance between the charge and the origin [83]. In the given experiment, the electric field potential was measured at a fixed distance at about 2 mm to the surface in the normal direction using a non-contact voltmeter. Since the non-contact voltmeter (Trek 884 Non-Contacting Electrostatic Voltmeter) has negligible error in measurement as the normal distance between the surface and voltmeter probe is increased, the distance can be treated as a constant, making the electric field proportional to the charge. Hence, the observations made using the non-contact voltmeter can be used as an indicator of the charge-to-mass ratio. In this study, the results are presented in the form of the differential electric field (DEF) compared for different cases. The case matrix is shown in Table 2.

## 3. Results

### 3.1. SEM Images of Electrostatic vs. Traditional Spray Deposition

**Figure 4:**
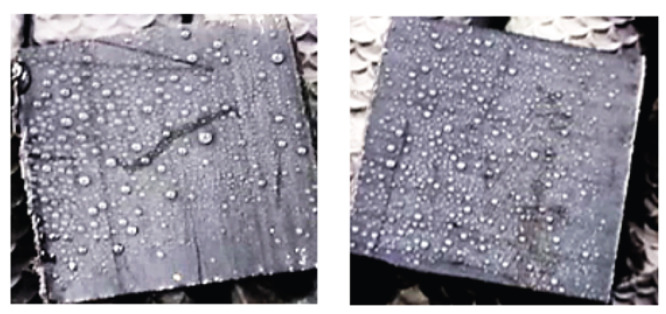
Camera image of Teflon surface for non-ESD (**left**) and ESD (**right**).

Figure 4 shows camera images of a Teflon sample coated using electrostatic spray and traditional spray. In case of electrostatic spray deposition, droplets are uniform in size and evenly spread over the surface area of the sample. For traditional spray deposition, non-uniform droplet size and distribution is observed.

**Figure A1:**
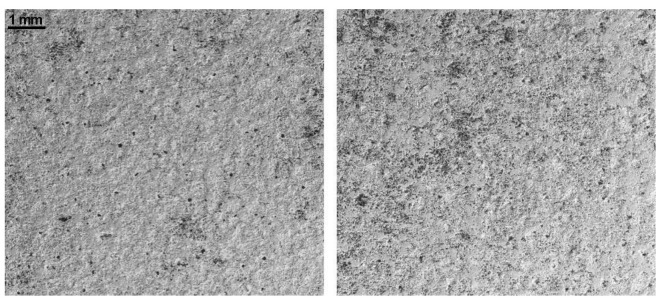
SEM image of stainless steel surface for non-ESD (**left**) and ESD (**right**) scaled to 1 mm.

After 24 h of application of Nanoxen on the surface samples, the presence of nanoparticles is observed and analyzed in the back-scattered images obtained under the scanning electron microscope. In Appendix A, Figure A1 shows back-scattered SEM images of stainless steel when sprayed under ESD and non-ESD conditions. It is observed that for both cases, the surface is completely covered with the nanoparticle solution in 10 s. We see a thick film of nanoparticles distributed throughout the metal surface. Similar results are observed in aluminum, copper and cast iron. There is dripping and thick layering of the nano-solution on these metal surfaces.

**Figure A2:**
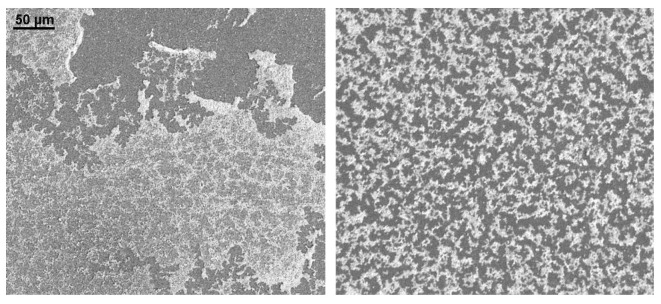
SEM image of polyethylene surface for non-ESD (**left**) and ESD (**right**) scaled to 50 µm.

**Figure A3:**
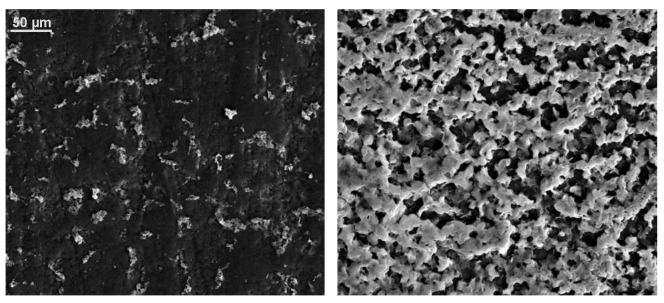
SEM image of Teflon surface for non-ESD (**left**) and ESD (**right**) scaled to 50 µm.

**Figure A4:**
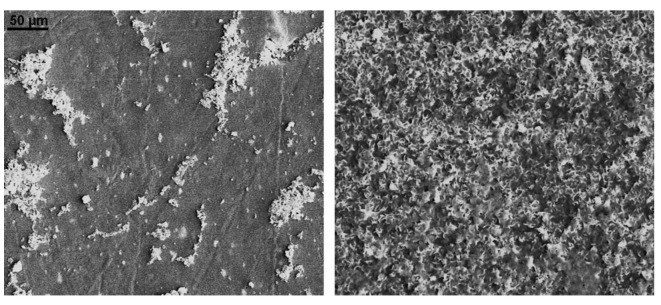
SEM image of PVC surface for non-ESD (**left**) and ESD (**right**) scaled to 50 µm.

**Figure A5:**
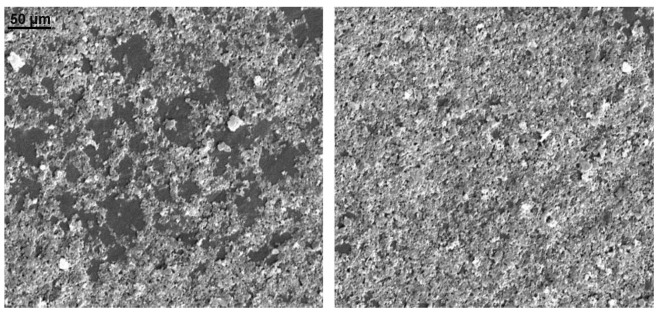
SEM image of polypropylene surface for non-ESD (**left**) and ESD (**right**) scaled to 50 µm.

**Figure A6:**
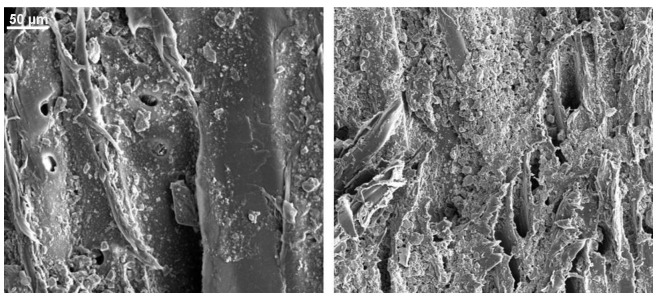
SEM image of wood surface for non-ESD (**left**) and ESD (**right**) scaled to 50 µm.

Plastics and polymers like acrylic, Teflon, PVC, and polypropylene show a distinct effect of ESD and non-ESD spray. The nanoparticles form a uniform layer with better surface coverage in case of electrostatic deposition, as seen in Appendix A Figure A2, Figure A3, Figure A4, Figure A5, respectively. Due to the highly porous nature of the sample substrate wood, the nano-particles are deposited in a non-uniform fashion for both ESD and non-ESD spray. However, more clustering and agglomeration is observed in non-electrostatic deposition, as shown in Appendix A, Figure A6. The results from SEM imaging suggest that the effective deposition of disinfectant nano-particles is obtained in case of the electrostatic spraying technique. It can be seen that different substrate types respond to the electrostatic spray in an inconsistent manner. These qualitative observations present the need to vary system parameters when spraying different types of substrate materials. The default setting of ion generator voltage, flow rate and nozzle size that is widely used in several disinfection applications currently might not be the most optimized set of parameters for safe and effective spraying. This could become a contributing factor to the spread of respiratory pathogens.

### 3.2. Effect of Varying Voltage, Nozzle Size and Working Distance

**Figure A7:**
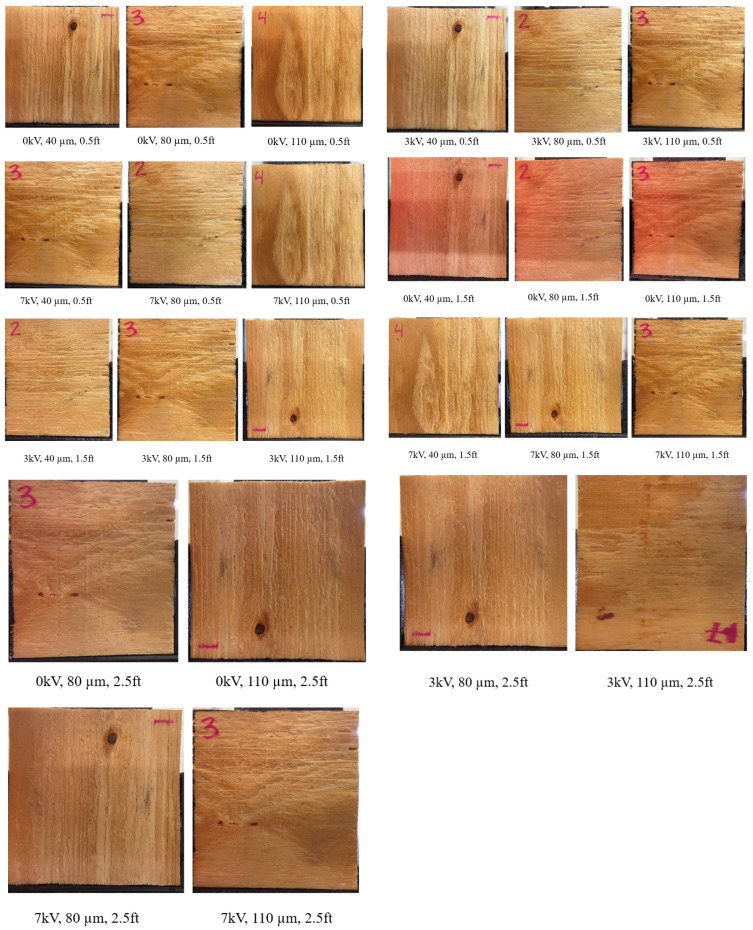
Wood: qualitative images of electrostatic deposition under various system parameters.

**Figure A8:**
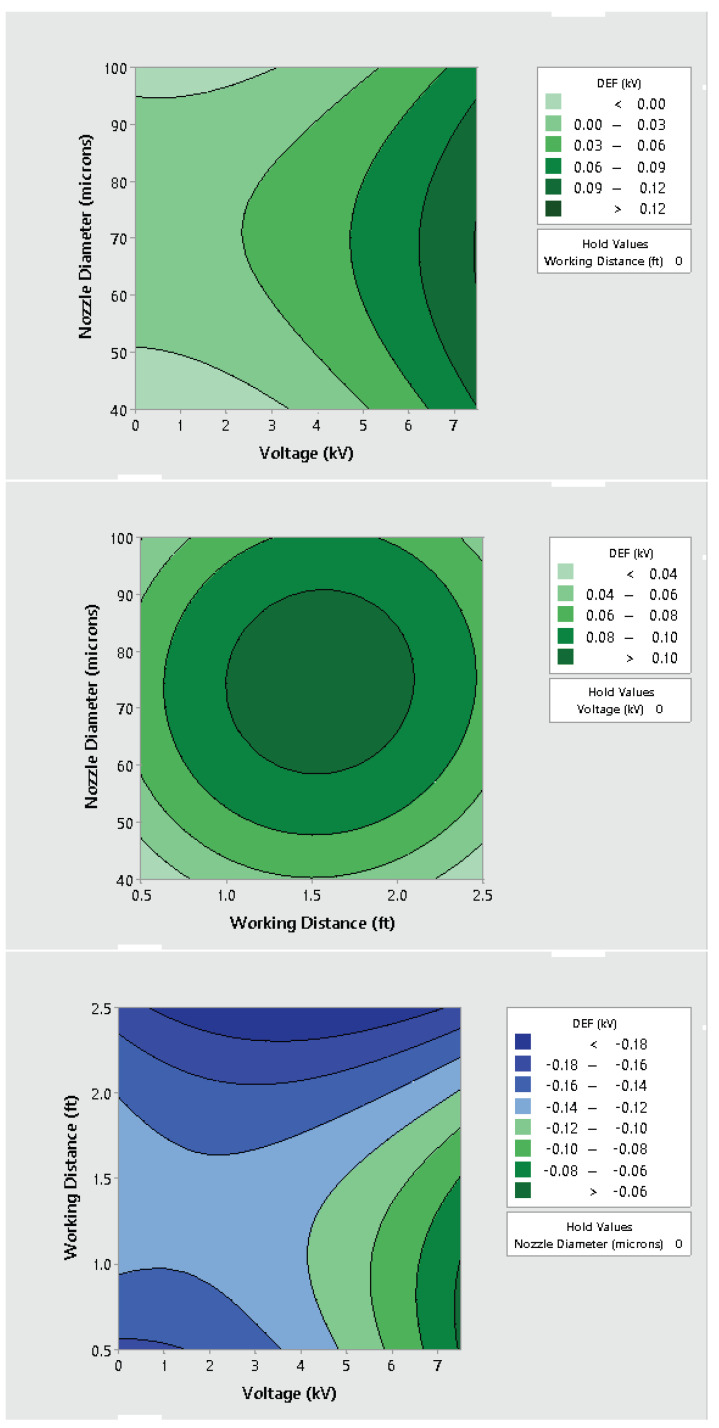
Wood: Quantitative representation of average differential electric field vs. working distance (feet), nozzle sprayer size (μm), and charge voltage (kV).

**Figure A9:**
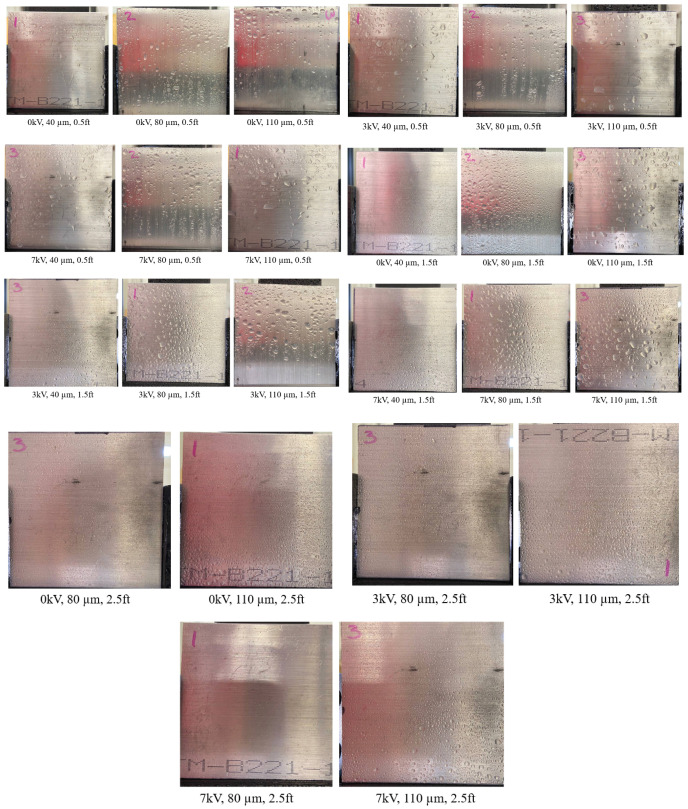
Aluminum: qualitative images of electrostatic deposition under various system parameters.

**Figure A10:**
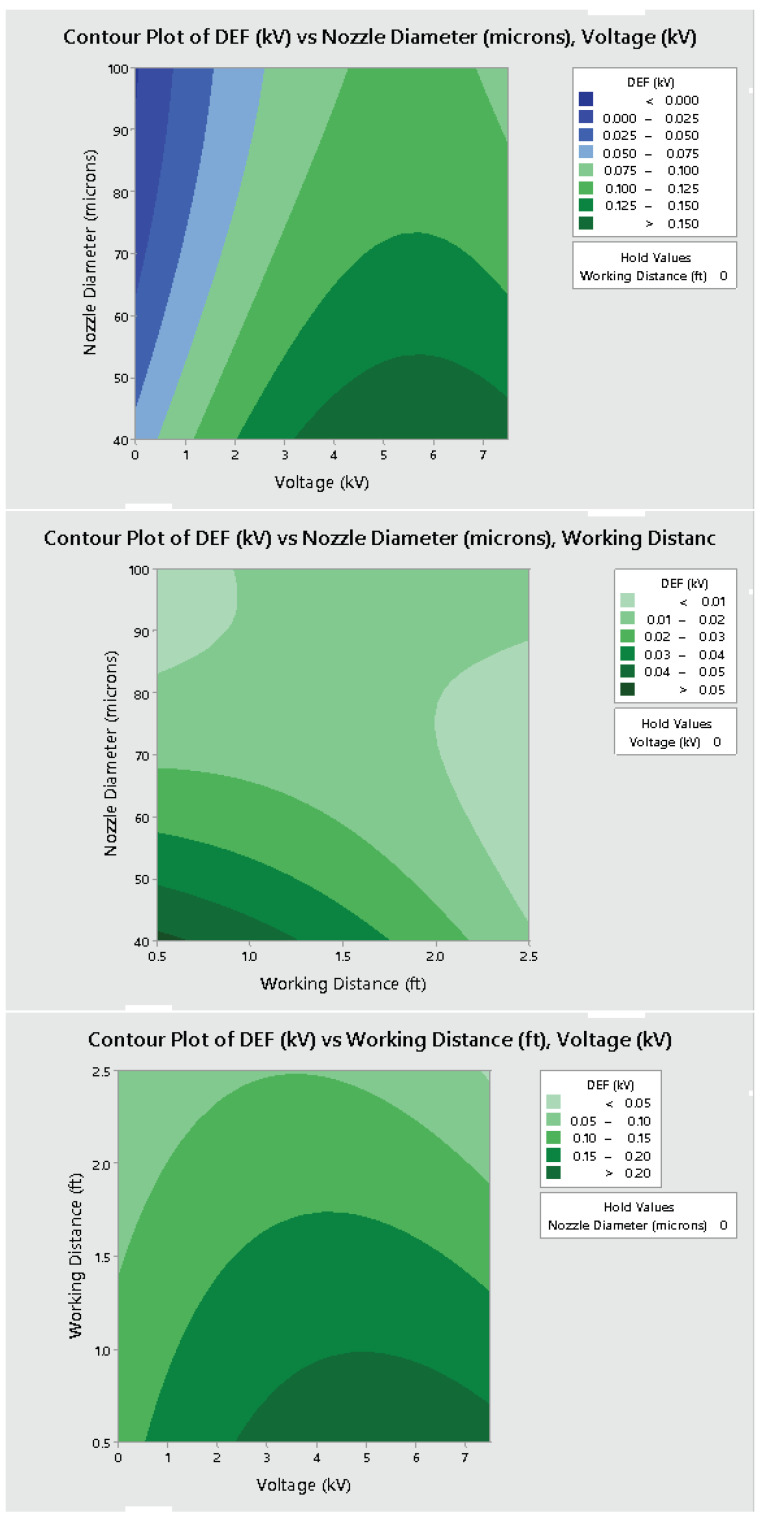
Aluminum: quantitative representation of average differential electric field vs. working distance (feet), nozzle sprayer size (μm), and charge voltage (kV).

**Figure A11:**
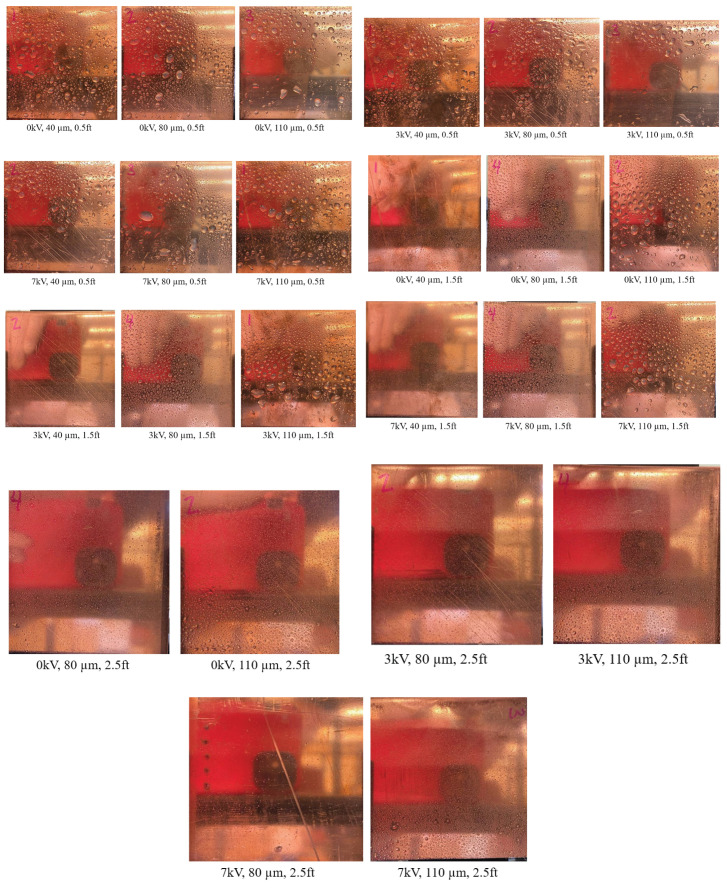
Copper: qualitative images of electrostatic deposition under various system parameters.

**Figure A12:**
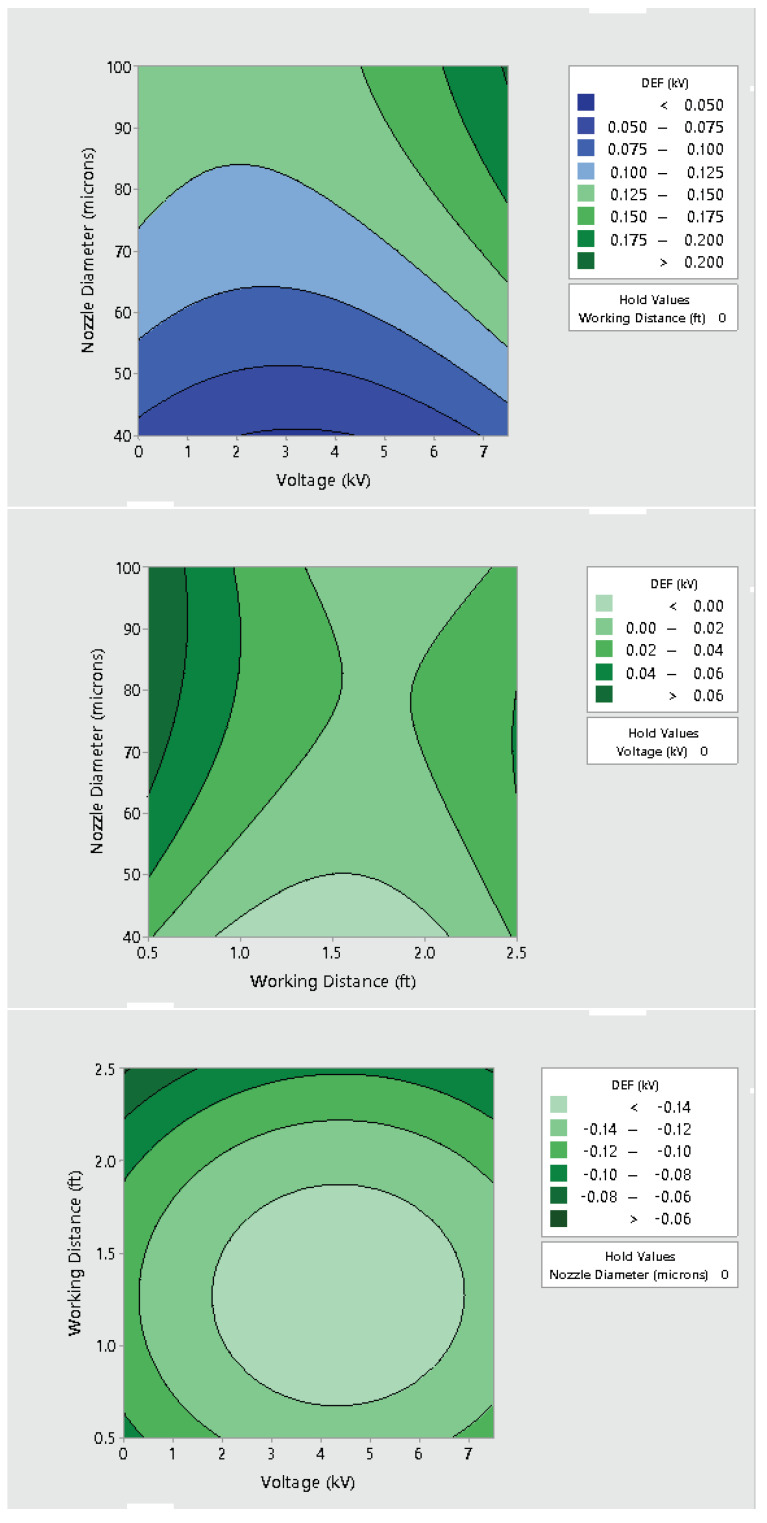
Copper: quantitative representation of average differential electric field vs. working distance (feet), nozzle sprayer size (μm), and charge voltage (kV).

**Figure A13:**
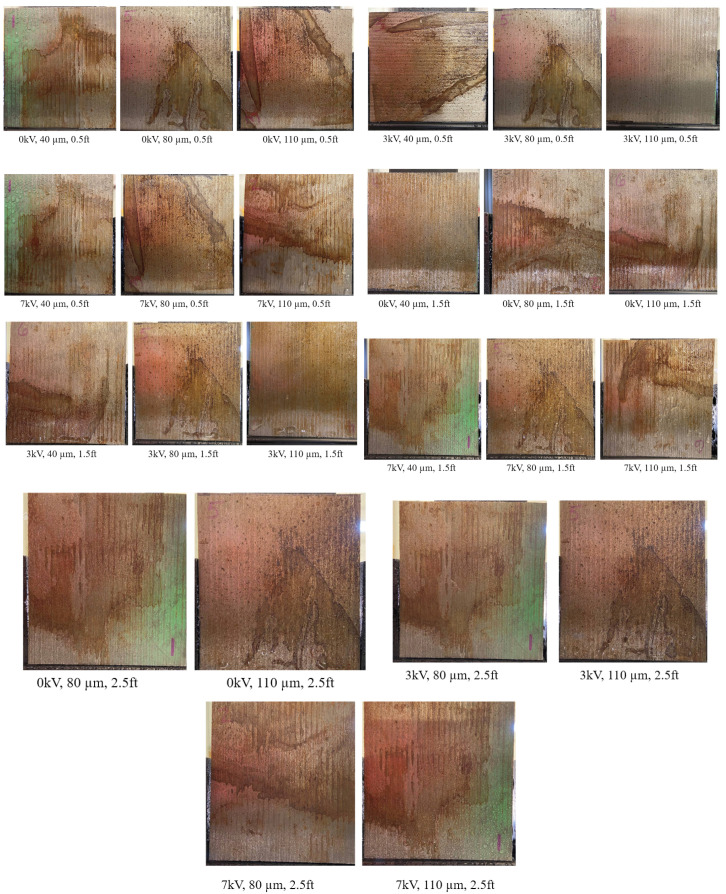
Cast iron: qualitative images of electrostatic deposition under various system parameters.

**Figure A14:**
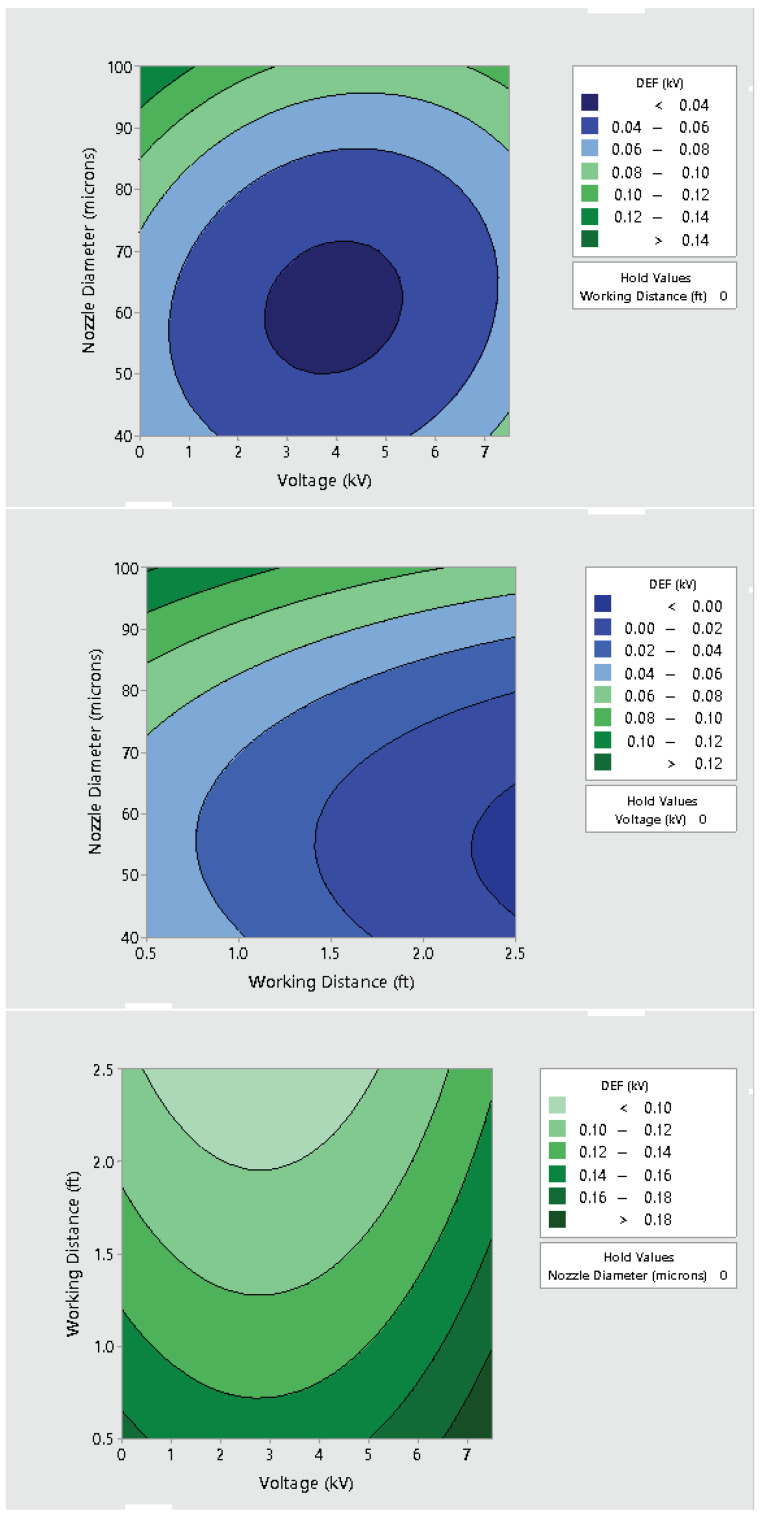
Cast iron: quantitative representation of average differential electric field vs. working distance (feet), nozzle sprayer size (μm), and charge voltage (kV).

**Figure A15:**
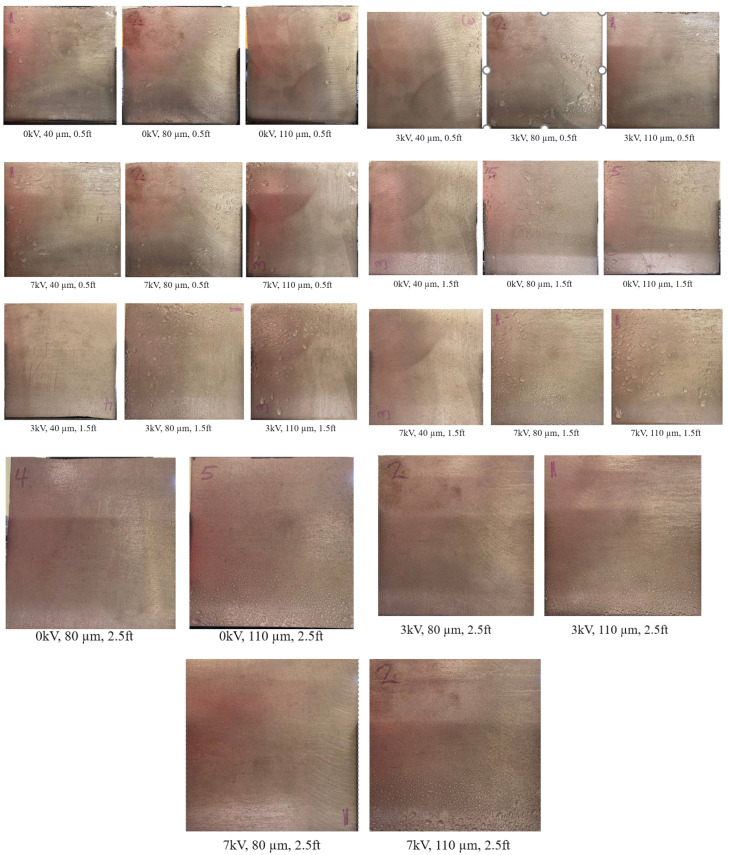
Stainless steel: qualitative images of electrostatic deposition at various system parameters.

**Figure A16:**
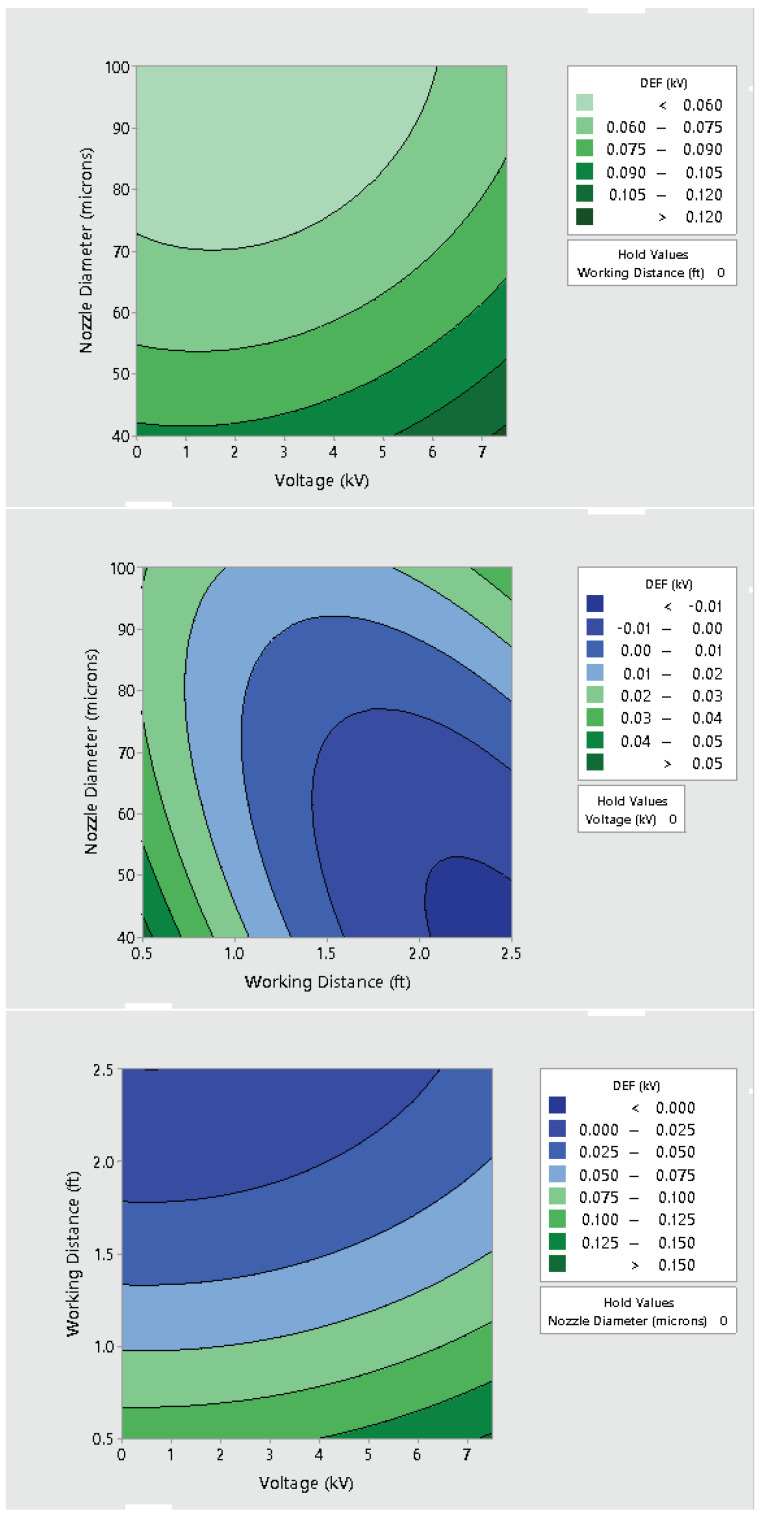
Stainless steel: quantitative representation of average differential electric field vs. working distance (feet), nozzle sprayer size (μm), and charge voltage (kV).

**Figure A17:**
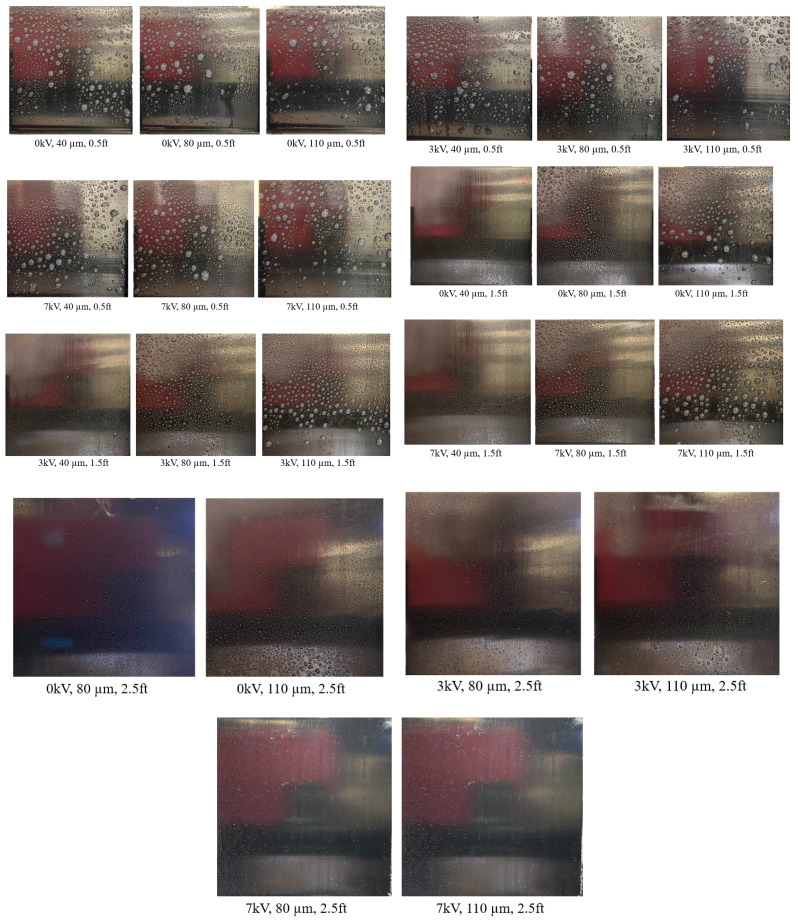
Polypropylene: qualitative images of electrostatic deposition under various system parameters.

**Figure A18:**
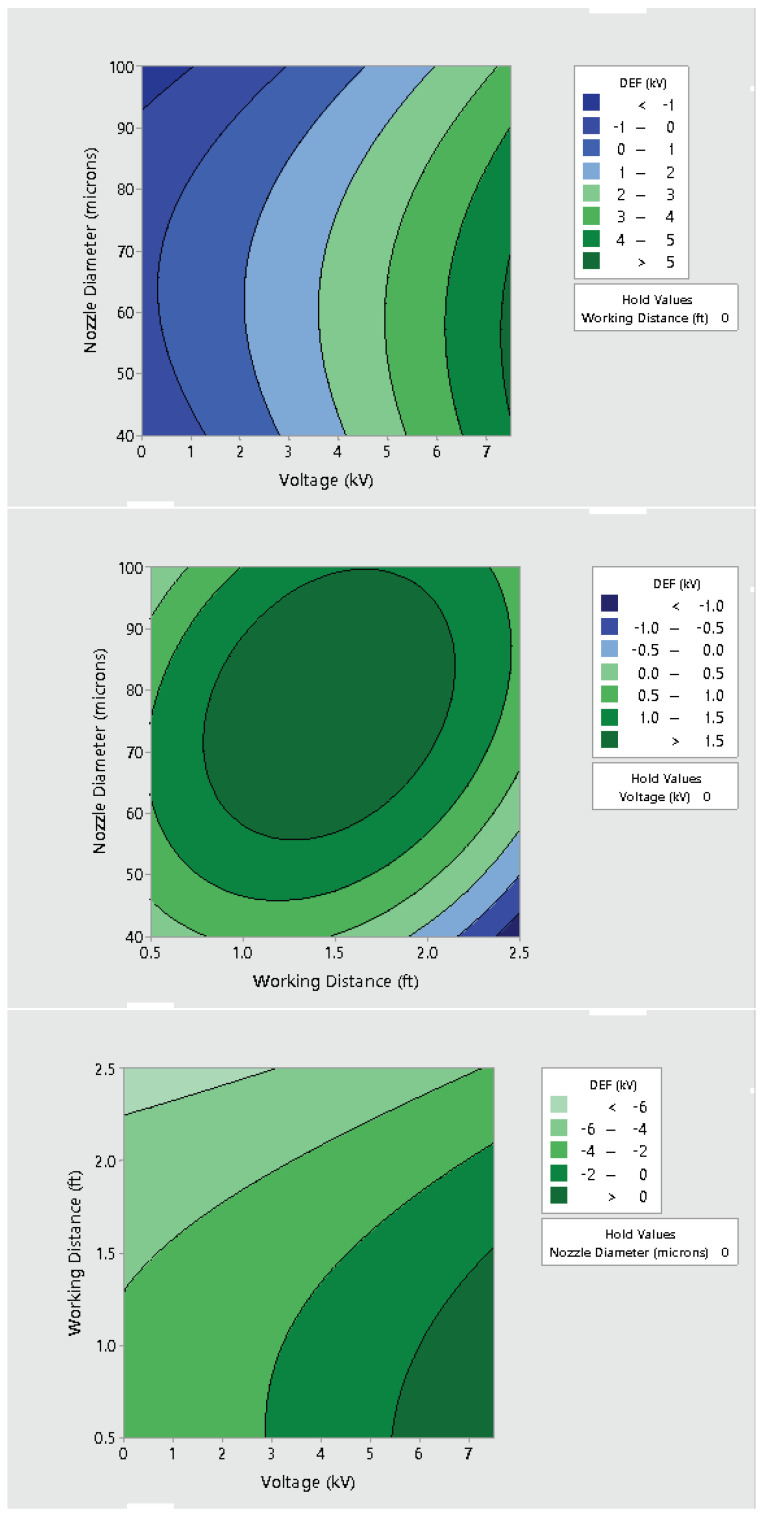
Polypropylene: quantitative representation of average differential electric field vs. working distance (feet), nozzle sprayer size (μm), and charge voltage (kV).

**Figure A19:**
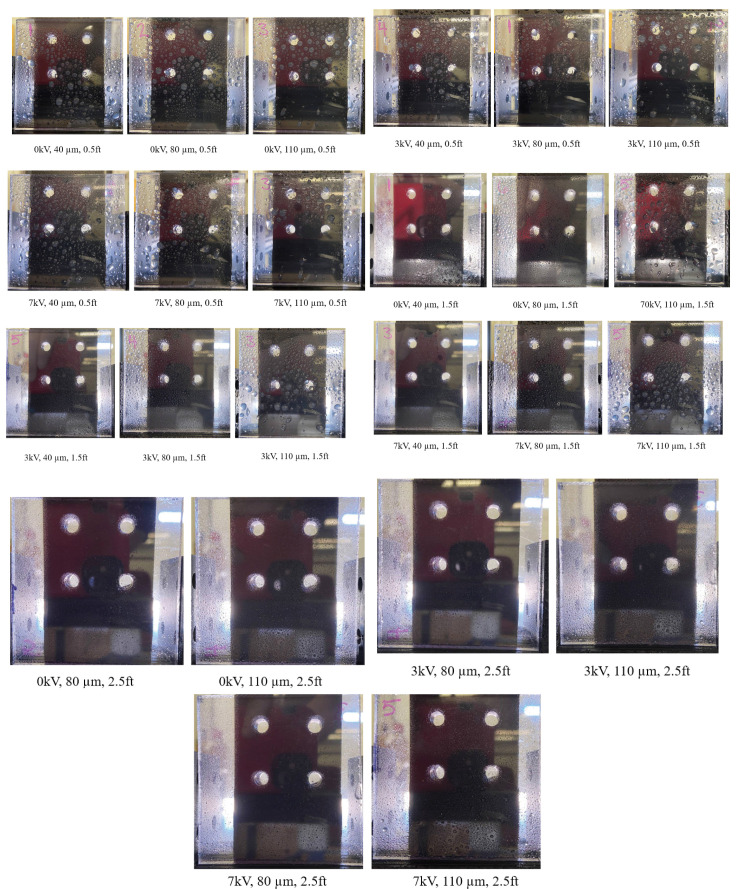
Polyethylene plastic: qualitative images of electrostatic deposition under various system parameters.

**Figure A20:**
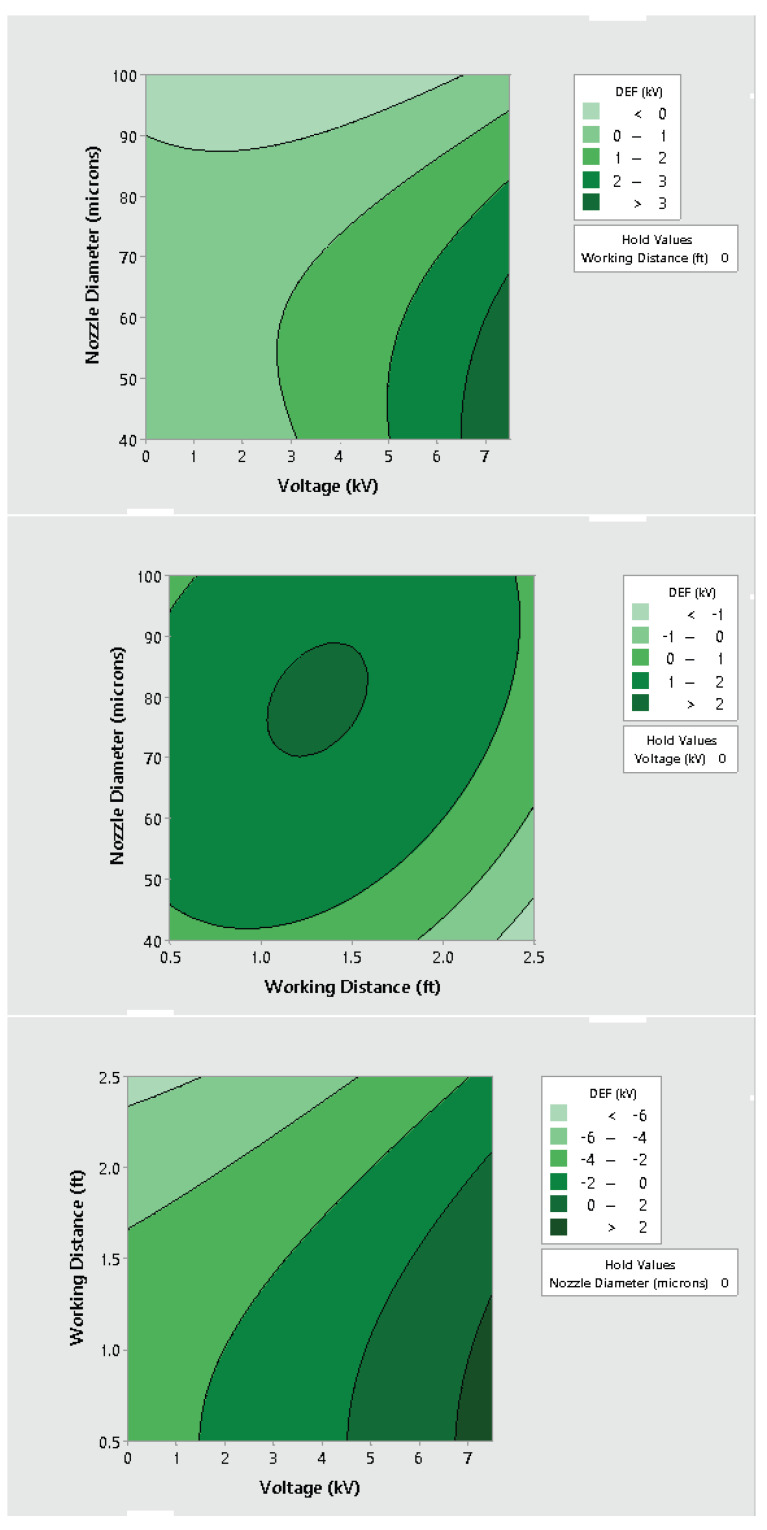
POLYETHYLENE PLASTIC: Quantitative representation of average differential electric field vs. working distance (feet), nozzle sprayer size (μm), and charge voltage (kV).

**Figure A21:**
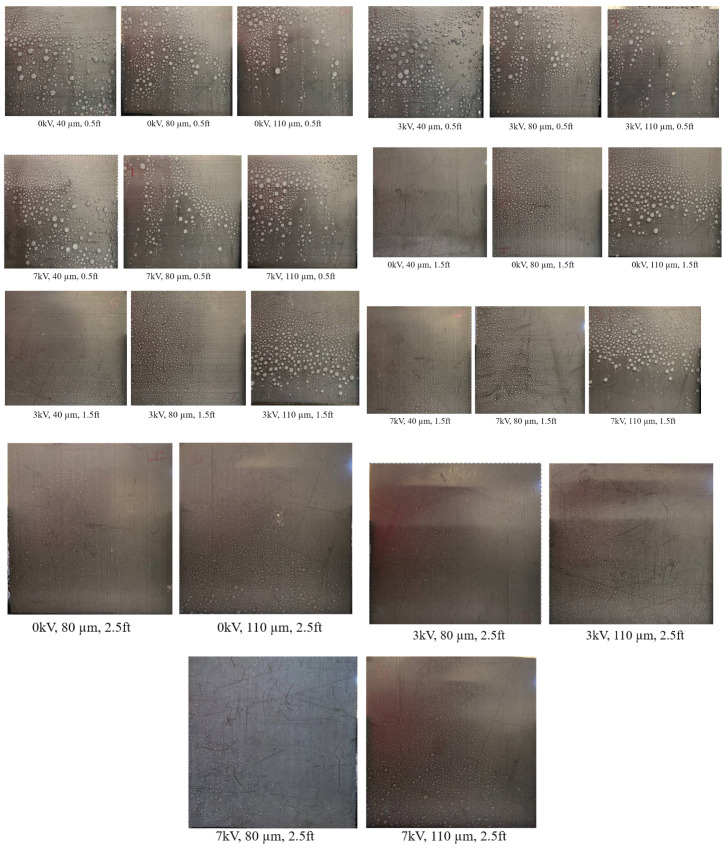
Teflon: qualitative images of electrostatic deposition under various system parameters.

**Figure A22:**
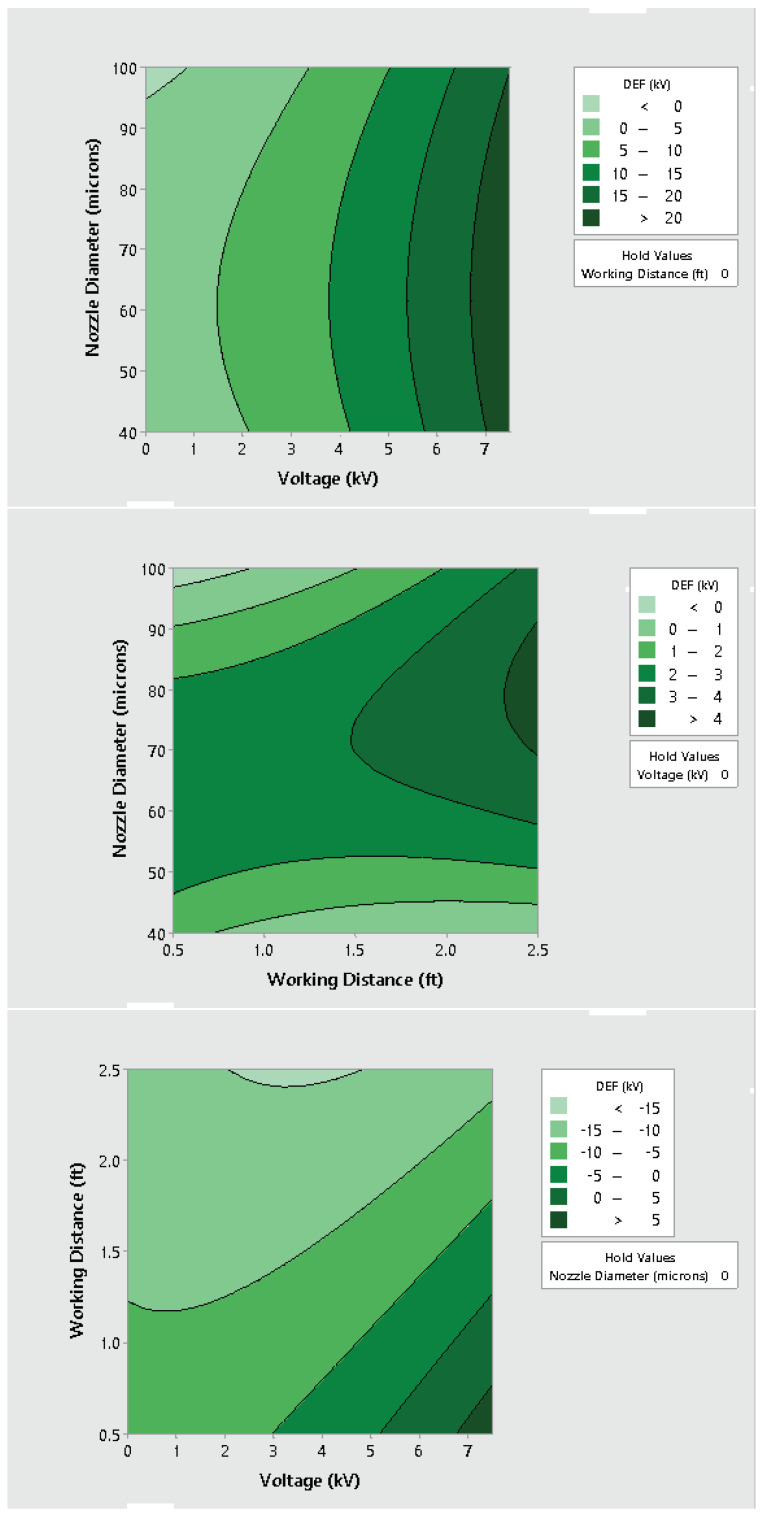
Teflon: quantitative representation of average differential electric field vs. working distance (feet), nozzle sprayer size (μm), and charge voltage (kV).

**Figure A23:**
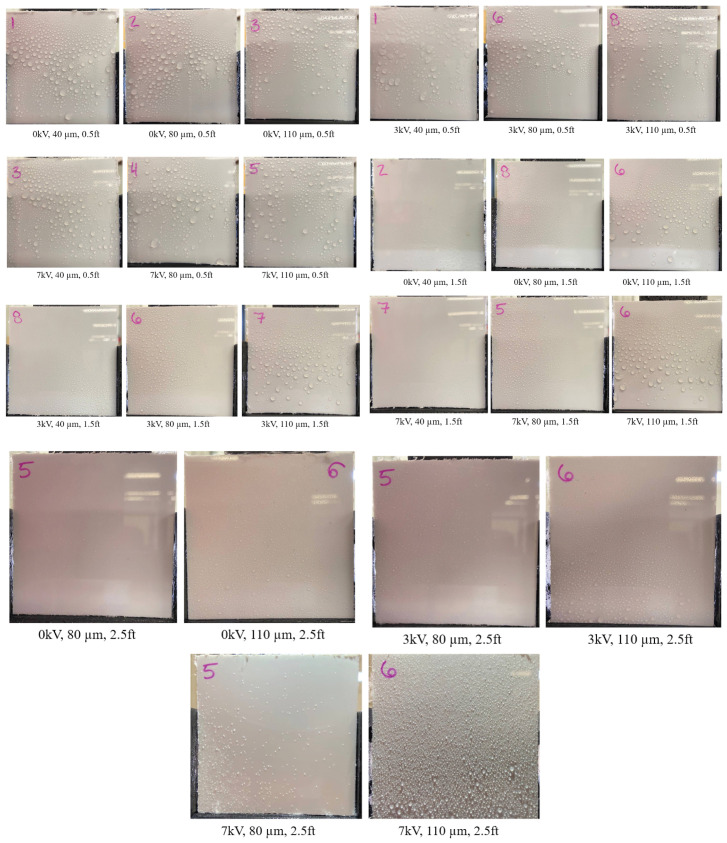
PVC: qualitative images of electrostatic deposition under various system parameters.

**Figure A24:**
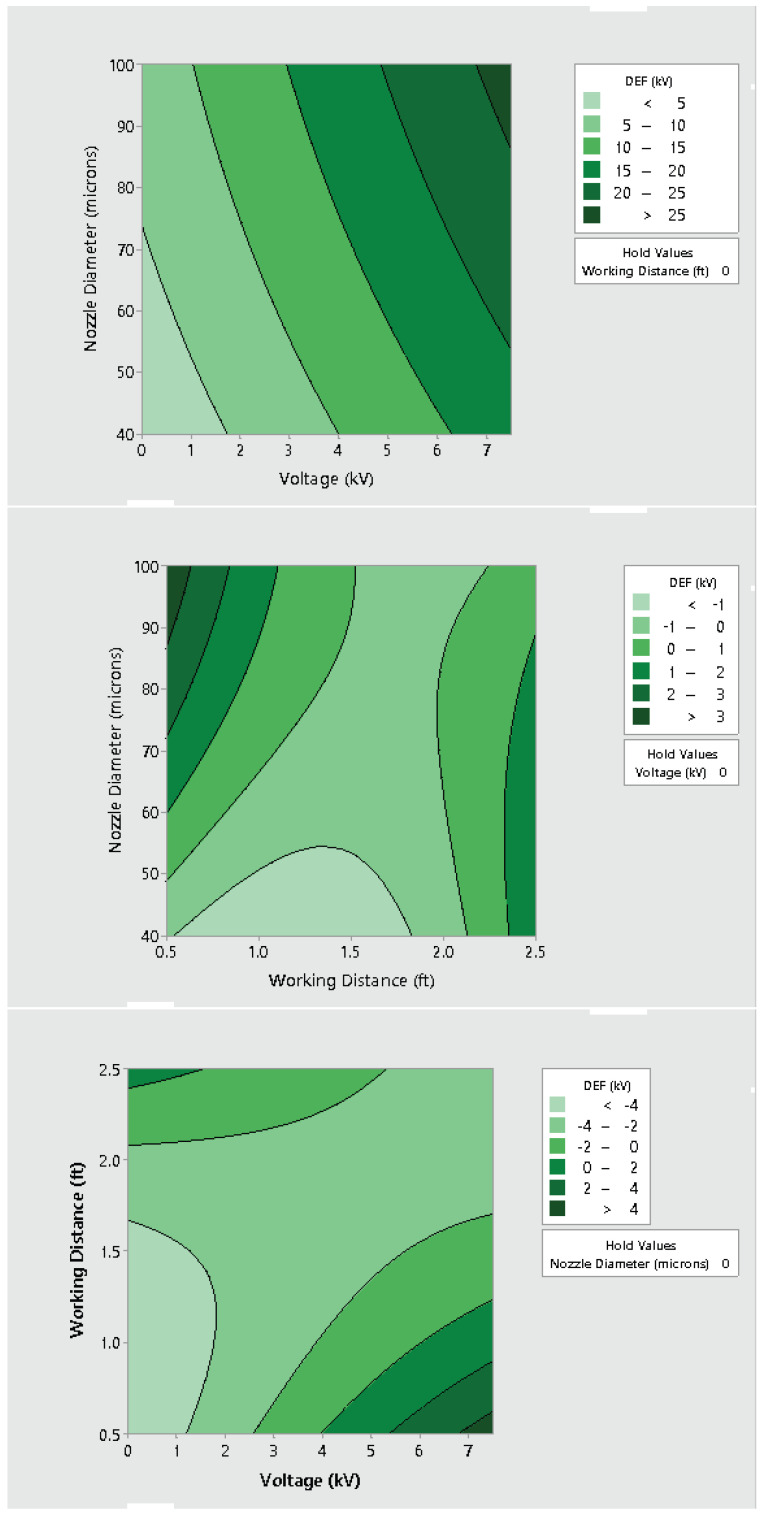
PVC: quantitative representation of average differential electric field vs. working distance (feet), nozzle sprayer size (μm), and charge voltage (kV).

**Figure A25:**
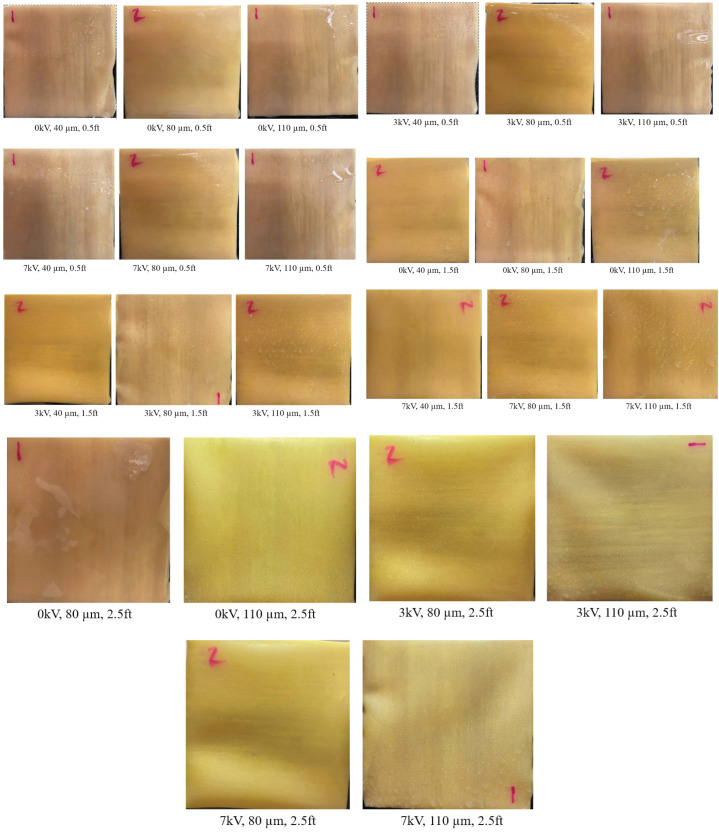
Rubber: qualitative images of electrostatic deposition under various system parameters.

**Figure A26:**
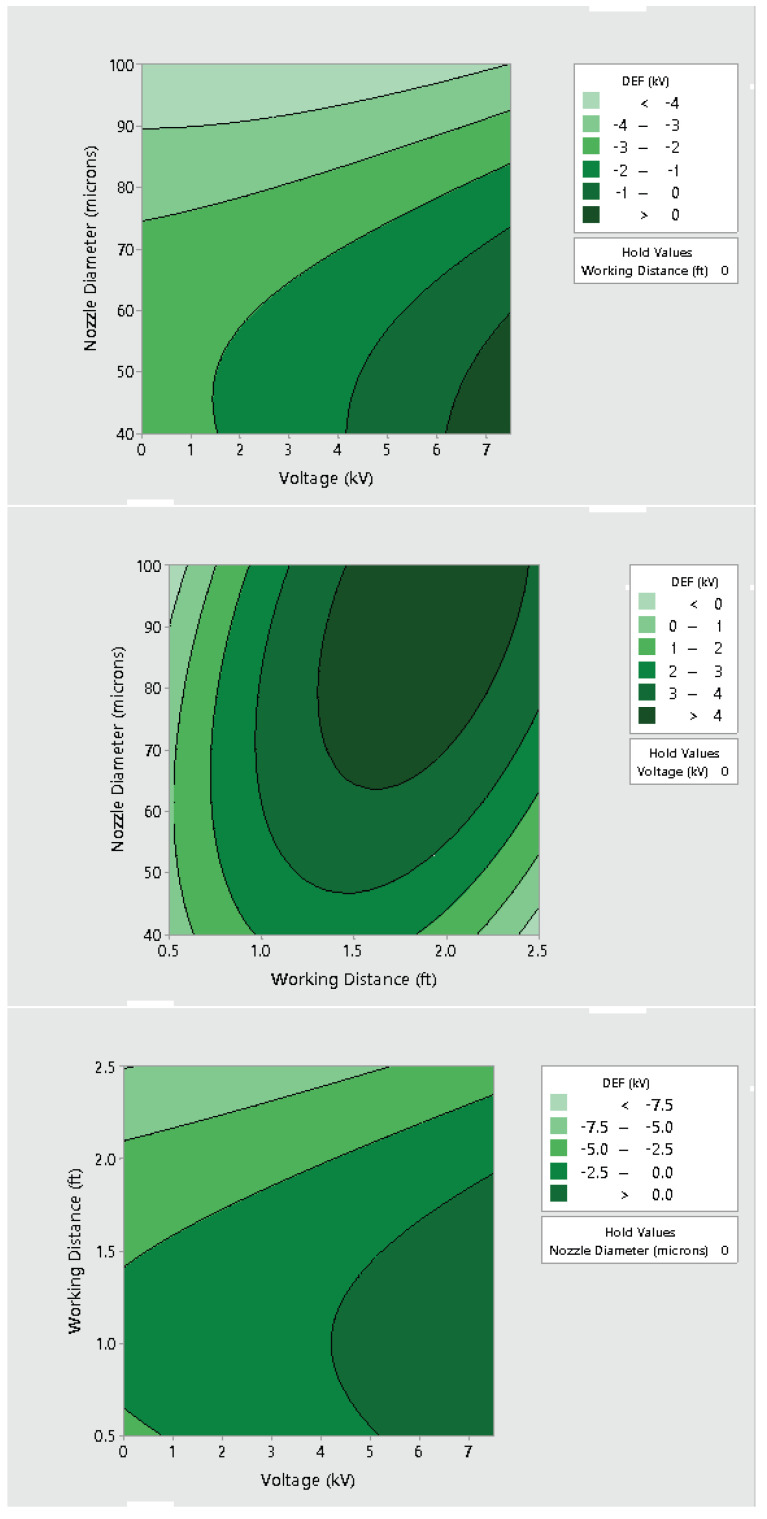
Rubber: quantitative representation of average differential electric field vs. working distance (feet), nozzle sprayer size (μm), and charge voltage (kV).

**Figure A27:**
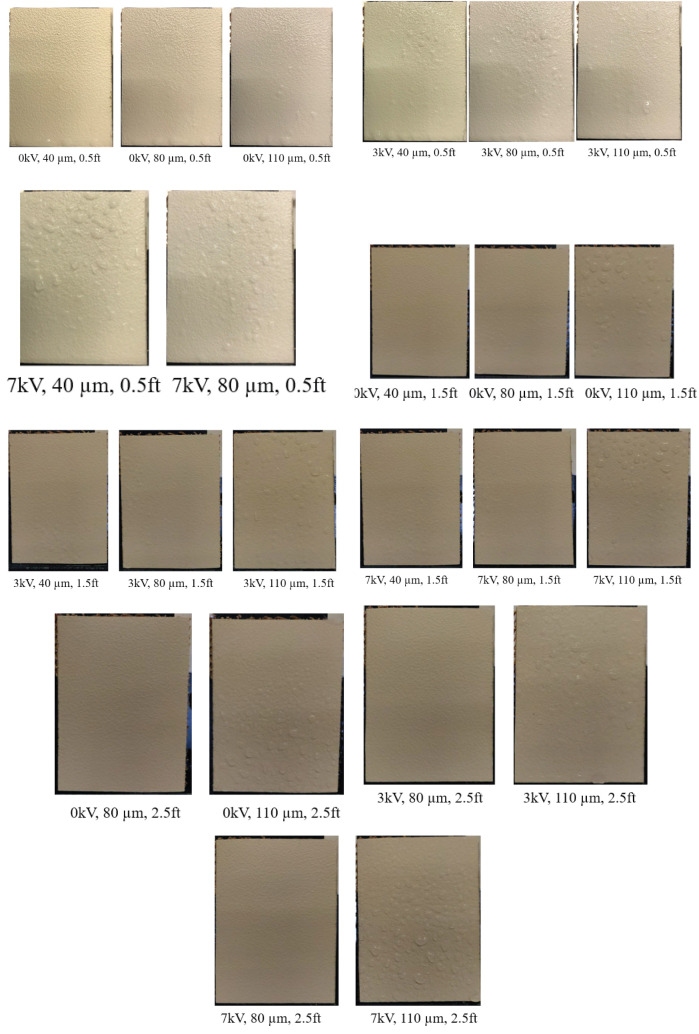
Aircraft sample-1: qualitative images of electrostatic deposition under various system parameters.

**Figure A28:**
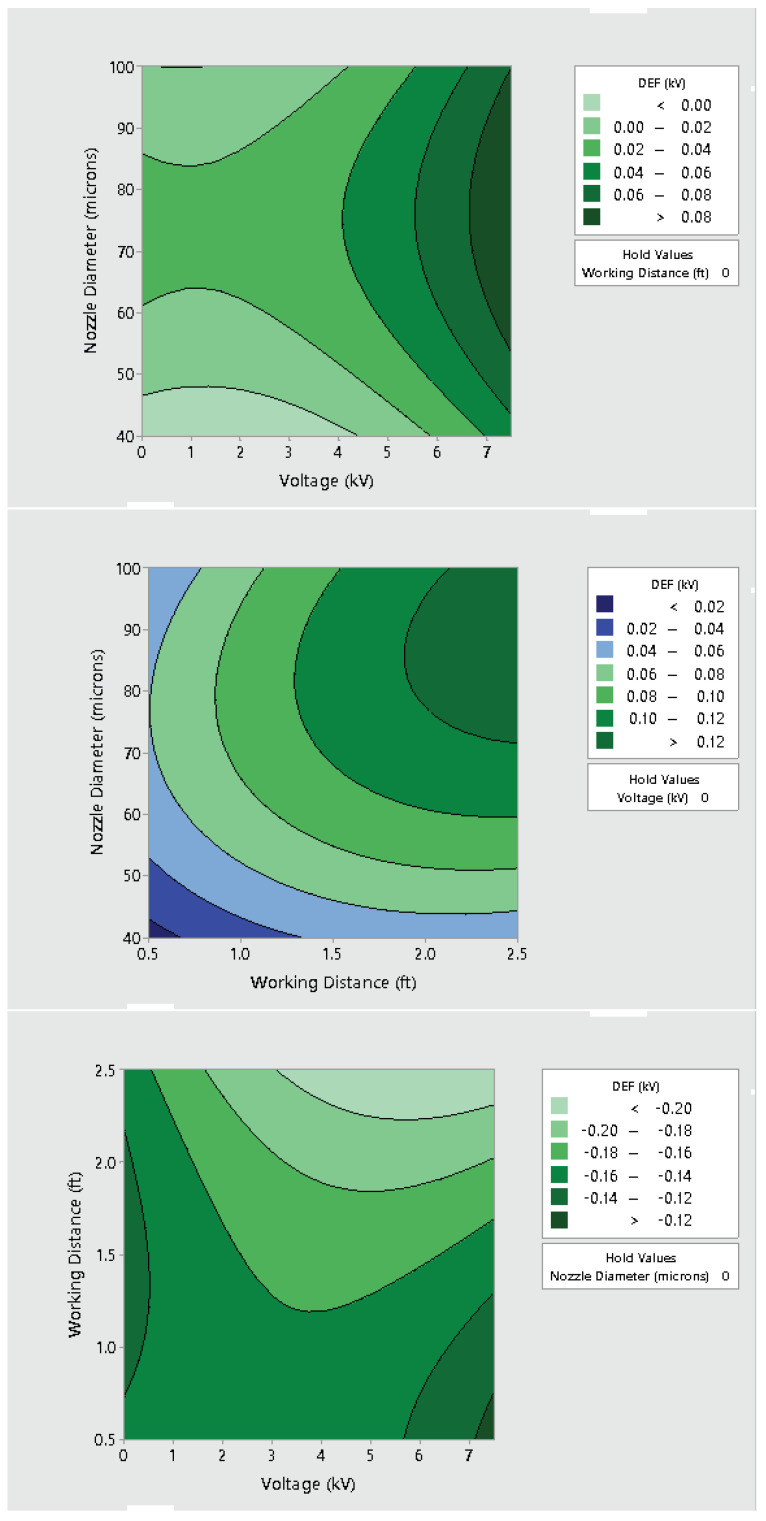
Aircraft sample-1: quantitative representation of average differential electric field vs. working distance (feet), nozzle sprayer size (μm), and charge voltage (kV).

**Figure A29:**
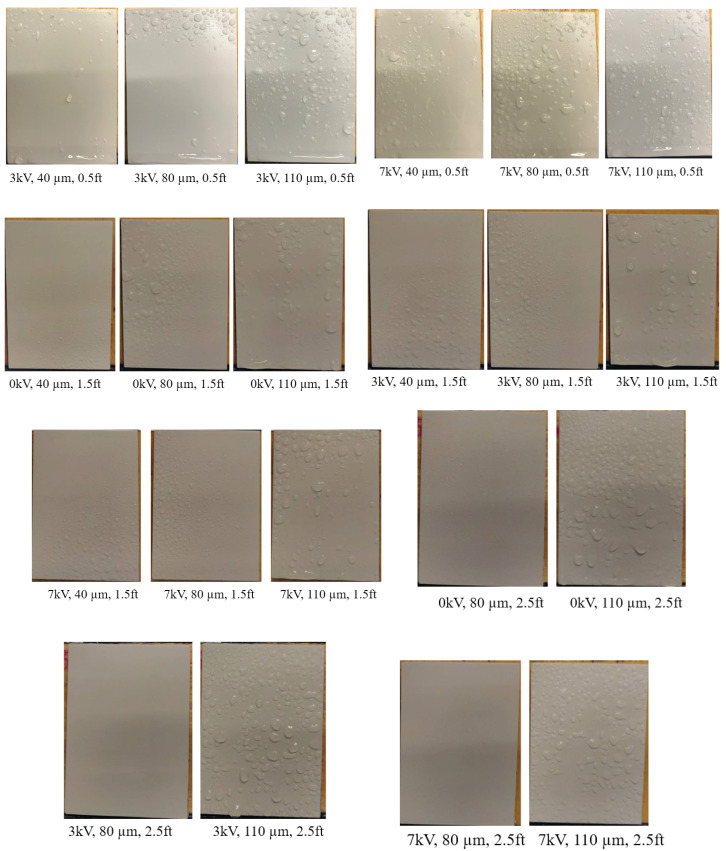
Aircraft sample-2: qualitative images of electrostatic deposition under various system parameters.

**Figure A30:**
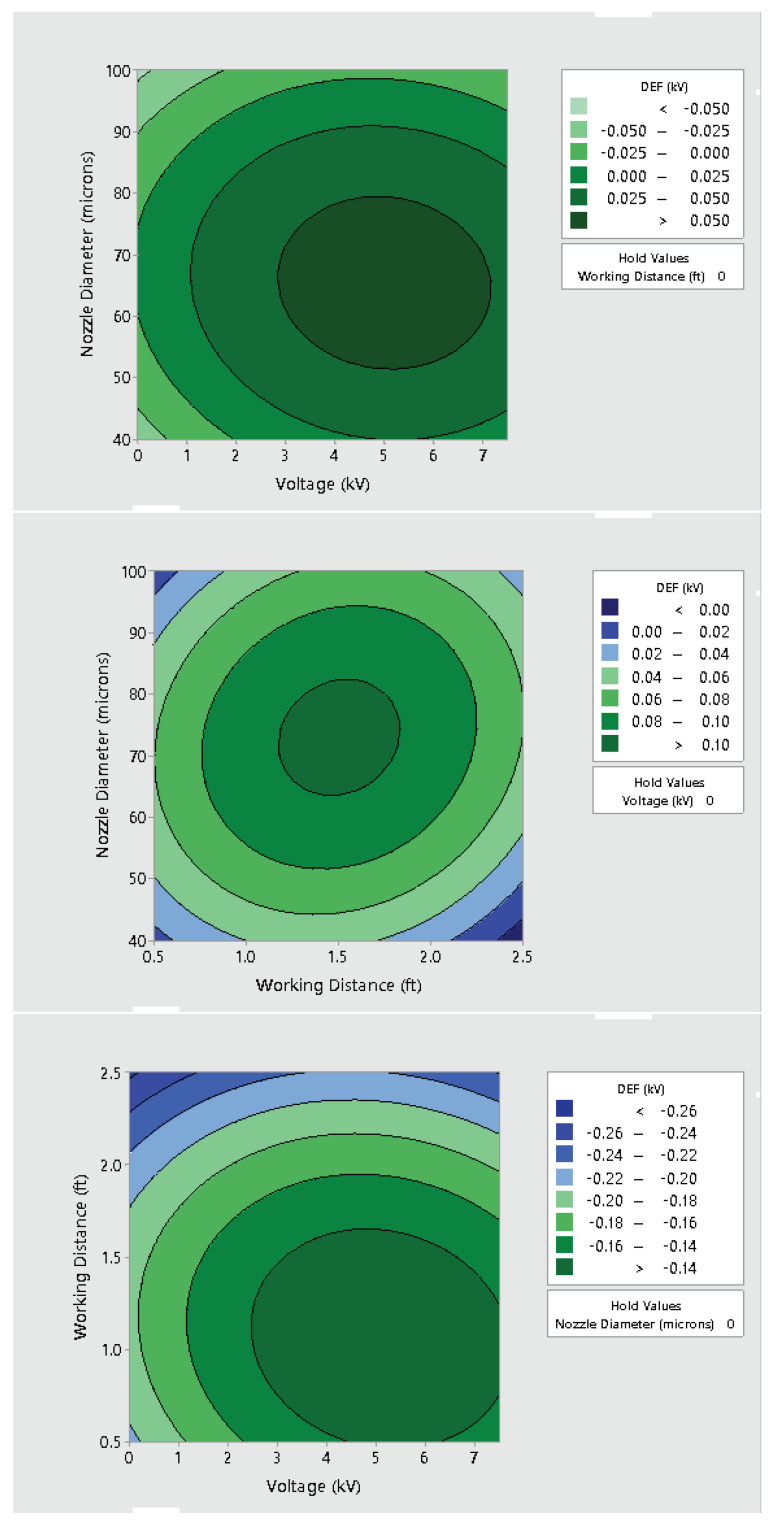
Aircraft sample-2: quantitative representation of average differential electric field vs. working distance (feet), nozzle sprayer size (μm), and charge voltage (kV).

Appendix B, Figure A7 shows the qualitative images of the substrate sample wood. It is observed that the surface becomes completely wet in 3 s of spraying and does not have any distinct droplets visible due to its porous nature. Dripping is observed. All the cases look qualitatively similar. The plots in Appendix B Figure A8 represent the quantitative variation and correlation of one system parameter with another based on the experimental data collected. It is observed that at around 1.5 feet of working distance, an 80 μm spray nozzle size and an ion generator voltage above 6 kV helps in attaining a high DEF (differential electric field). This corresponds to a better charge-to-mass ratio and, in turn, ensures better spread of the nano-precursor solution. For the aluminum substrate, the qualitative data are shown in Appendix B, Figure A9. It is observed that spraying at a distance of 1.5–2.5 feet at an 80 μm spray size ensures proper area coverage as well as the presence of uniformly sized droplets on the surface. The quantitative analysis of the surface plots shown in Appendix B Figure A10 shows that the effect of voltage is negligible because the metal surface does not retain much charge on its own. Here, we observe a contrast to the qualitative results. The best region for effective spray outcome is in the ranges of 2–7 kV, 40–60 μm and 0.5–1.5 feet of working distance. Like other metallic substrates, it is qualitatively observed that copper does not exhibit any major improvement in spray effectiveness because of changes in the ion generator voltage. Appendix B, Figure A11 shows the qualitative images obtained after spraying. However, changing the working distance and nozzle diameter shows a significant difference. The uniform spread of the nanodisinfectant is observed at an 80 μm nozzle setting and 1.5 feet working distance. Quantitative results in Appendix B, Figure A12 show that higher DEF is obtained at an 80 μm nozzle diameter and 2.5 feet of working distance independent of the voltage setting. For cast iron, qualitative images are given in Appendix B, Figure A13. No distinct droplets are observed on the surface. Most of the disinfectant drips off the surface. There are large droplets that mostly accumulate on the lower end of the vertical face. Plots in Appendix B, Figure A14 show that the value range of DEF is almost negligible. It is observed that a high nozzle spray diameter results in higher DEF relative to other conditions. Appendix B Figure A15 presents the qualitative images for the substrate stainless steel. A non-uniform droplet distribution on the surface is observed for all cases. Additionally, droplets accumulate at the lower half of the surface. This is caused because of dripping of the nanodisinfectant solution. From plots in Appendix B, Figure A16, it can be observed that the value range of DEF is almost negligible, similar to other metallic substrates. Lower values of working distance and and nozzle spray size results in higher DEF. For the substrate material polypropylene, Appendix B, Figure A17 presents the qualitative observations. At a working distance of 1.5 feet and an 80 μm nozzle spray setting, a uniform droplet distribution is observed over the surface area independent of the voltage. The plots in Appendix B, Figure A18 show agreement with the qualitative results at a high ion generator voltage setting. The qualitative observations for the substrate polyethylene plastic are given in Appendix B, Figure A19. At a working distance of 1.5 feet and a nozzle diameter of 80 μm, a uniform droplet distribution is observed on the surface. This is well in agreement with the quantitative data represented as surface plots in Appendix B, Figure A20 for higher ranges of the ion generator voltage. For the substrate material Teflon, Appendix B, Figure A21 presents the qualitative data. At a working distance of 1.5 feet and an 80 μm nozzle spray size, as well as at a working distance of 2.5 feet and a 110 μm nozzle setting, a uniform spread and droplet distribution are observed. From surface plots in Appendix B, Figure A22 it can be observed that a higher range of ion generator voltages and a range of nozzle settings between 50 and 80 μm are effective in terms of attaining high DEF. Appendix B, Figure A23 shows that the substrate PVC has a similar qualitative nature as Teflon after spraying. Quantitatively, as shown in Appendix B, Figure A24, either a high ion generator voltage with a low working distance or a low ion generator voltage and high working distance are a more effective system setting for optimized spraying. On the rubber surface, no distinct droplets are observed, as shown in Appendix B, Figure A25. Wetting and dripping is observed, except at the highest nozzle diameter size and working distance. Quantitatively, as shown in Appendix B, Figure A26, all three system parameters at the higher ranges of values help in attaining maximum DEF. For the samples from the aircraft cabin, the qualitative images for the substrate aircraft sample 1 are shown in the Appendix B, Figure A27. Large, non-uniform droplets and dripping are observed in most cases. From the quantitative results shown in Appendix B, Figure A28, it is observed that for a working distance between 1.5 and 2.5 feet, a nozzle diameter between 60 and 100 μm as well as a higher range of the ion generator voltage setting results in maximum DEF values. For the substrate aircraft sample 2, Appendix B, Figure A29 shows that effective spraying is obtained at an 80 μm nozzle setting and 1.5 feet working distance. This is in agreement with the quantitative results presented in Appendix B, Figure A30 for high ion generator voltages ranging between 3 and 7 kV.

## 4. Discussion

COVID-19 pandemic has brought to the forefront an urgent need for strategic disinfection practices so as to curb the spread of common respiratory viruses through fomites. Our study has brought to light the advantages of using the electrostatic deposition of a nanoparticle-based disinfectant for effective disinfection practices and specified parameters that would best suit the spraying conditions given the target surface material. Through discussions with leading electrostatic sprayer agencies (https://www.victoryinnovations.com/, accessed on 2 June 2022), we ascertained that there is a need in the industry to understand the disinfection of hard and vertical surfaces. Most companies conducting disinfection in closed spaces for example, an airplane cabin, have open questions about the sprayer settings that are best suited for surfaces in particular environments. They have been using the default settings or the “most obvious” settings. This can lead to improper disinfection. Previous studies are more focused on classical disinfection strategies when it comes to disinfection of surfaces. Classical strategies mostly include the use of chemical disinfectants like hypochlorite, peroxymonosulfate, alcohols, quaternary ammonium compounds, and hydrogen peroxide [57,84]. They also include ultraviolet (UVC) irradiation, solar irradiation and heat treatment [85]. Castaño et al. [85] highlighted that chemical disinfectants can be evaluated based on suspension and carrier tests. Prior carrier tests have been performed using stainless steel surfaces, which may not reflect the disinfectant effectiveness on other fomites that have different surface properties. They show that UVC-based disinfection takes a very long time in the disinfection process and poses a high health risk to exposed individuals. Heat treatment is an effective method, as high temperatures can help in virus inactivation by damaging the protein capsid; however, it cannot be used for everyday purposes. There are several limitations to the use of chemical-based disinfectants, such as harmfulness, its corrosive nature and bacterial resistance [46]. Advanced disinfection technologies, such as self disinfecting materials [85], anti-viral coatings [57], automated procedures [57,86], nanoparticle-based disinfectants [46] and electrostatic spraying methods [26,87], are becoming famous strategies due to their long-lasting and high-precision disinfection. Although such studies highlight new technologies that can enhance the effectiveness of existing methods, they lack comprehensive data that can help in a practical directive solution to the rapidly growing need for disinfection of highly touched fomites. Lauritano et al. [88] presented a literature review of 11 articles, wherein most studies proposed the use of alcohol-based disinfection agents at different concentrations as a biocidal agent against coronavirus. The review also discussed the use of chlorine-containing disinfectants as well as UVC for decontamination. However, they highlighted that there is a need for more specific measures that can suit particular environmental settings. Chen et al. [89,90] presented different strategies for effective disinfection in indoor and outdoor settings. They highlighted the use of an electrostatic sprayer as an emerging technology for effective disinfection as well as the use of different categories of disinfectants. They also discussed the role of high-touch surfaces in constructing cleaning procedures. However, none of these studies defined a unique set of methods (spraying technology, disinfectants) and parameters that can help in creating an optimized cleaning strategy for frequently and highly touched surfaces. Our study is focused on solving this problem and proposes the use of electrostatic spraying with a nanoparticle-based disinfectant solution. We define the electrostatic spray system parameters depending on the type of target material as well as show the use of nanoparticles as a disinfectant. Our findings suggest that the use of nanoparticles with electrostatic deposition can be highly efficient and long lasting, especially for hard-to-reach surfaces. We also show that there is a need to change the existing disinfection protocols in indoor and outdoor settings.

The surfaces selected for our study can be categorized as metals, plastics and wood. These surfaces are composed of different material properties that influence stable virus survival, including, for example, porosity, wettability and inherent anti-microbial properties. Studies suggest that porous surfaces are easier to disinfect as compared to non-porous surfaces [21,91]. Based on our findings, wood is relatively porous with low wettability and hence shows a non-uniform distribution of nanoparticles under SEM imaging. It gets wet very easily without any distinct droplet distribution and causes dripping of the disinfectant solution. Most metallic and plastic surfaces used in this study are non-porous, smooth surfaces with higher wettability, which affects the nature of droplet deposition and distribution when sprayed using the nano-disinfectant and electrostatic spraying method. Non-porous and hard surfaces have always been a challenge due to higher and longer viral retention as well as difficulty in uniform spread of disinfectant solution due to dripping. Depending on the range of the spray system parameters, some cases show robust and uniform droplet distributions for each metal and plastic sample. The non-conductive nature of most metals results in fast decay of the charge and hence, some ambiguities are observed between the qualitative and quantitative results. From the qualitative observations, the samples from the aircraft cabin pose a challenge in defining a set of parameters due to the lack of any case with uniform droplet distribution. Rubber, as a material, is hydrophobic as well as porous in nature. This influences the droplet distribution; hence, wetting and dripping are observed for a large range of parameters. It can be seen that the varying nature of surfaces and how they respond to the spray makes it imperative that a technology like electrostatic deposition is used so as to ensure the deposition of nanoparticles enhanced by the presence of the charge. One of the major advantages of using a nanoparticle-based solution in this study is that it helps in visualization through SEM imaging. From the observations, it can be stated that the best set of system parameters when using electrostatic deposition technique for disinfection are a nozzle spray setting of 80 μm, an optimum spray working distance of 1.5 feet from the substrate and 3–7 kV of ion generator voltage as compared to using the consistent default settings (40 μm nozzle size and 7 kV of ion generator voltage) that are currently used in the industry for all types of surfaces. Through this study, we have proposed the following novel strategies and observations:

- Using the electrostatic deposition of a nanoparticle-based solution can be a highly efficient strategy for disinfection. Previous studies have highlighted the exclusive use of both of these technologies; however, we have tried to show how the combination of both can play an important role in future disinfection strategies;
- Electrostatic spray system parameters play a crucial role in the effective spread of the disinfectant on the surfaces. The uniform spread of nanoparticles can be assured and highly optimized by tuning the controllable system settings as shown in this study;
- Each target substrate is unique due to the surface properties and environmental factors. It is very crucial to take these into account when designing a strategy for disinfection, as shown in this study. This can be very effective in ensuring proper decontamination as well as in automating the process.

This study can be improved by comparing the results for nanoparticle disinfectants with the use of chemical disinfectants, especially by studying their lasting effects. The use of a surrogate virus to create contaminated sample surfaces can help in determining the effectiveness of this proposed disinfection strategy. The authors aim to further investigate this study and make it more robust.

## 5. Conclusions

Electrostatic spray deposition has been a well-known method for coating and spraying in many industries, such as the agriculture industry, food industry, and automobile industry. In 2019, when the COVID-19 pandemic started, causing lock downs, this method of disinfection became popular and well-recognized for its properties, such as its uniform, regular and thin coating with maximum surface area coverage. However, there was a lack of research that could show the effectiveness of this method on different surface materials that are commonly present and frequently used in indoor and outdoor spaces. For example, some companies presently conduct aircraft cabin disinfection using a 40 μm nozzle size and 7 kV of ion generator voltage for spraying disinfectants. This often results in dripping of the disinfectant solution, which escalates the challenges against safe and adequate disinfection. Our study highlights a strategic disinfection methodology by showing the use of an electrostatic nebulizer with a nano-engineered disinfectant solution for reliable and effective disinfection. Scanning electron microscopy is used to support the idea of using electrostatic deposition of nanoparticles for fighting pathogens as well as to observe the response of different surface materials to the proposed spraying methodology. An experiment is performed to explore the possibility of using different system parameters for electrostatic decontamination. Varying the system parameters such as the spraying working distance, nozzle diameter spray size and ion generator voltage allows the effect of electrostatic spray deposition to be tested on common, frequently touched surfaces. These surfaces are selected based on the fact that they have been found to remain contaminated and sustain contagious viruses longer. The observations from this experiment show that a nozzle spray setting of 80 μm, an optimum spray working distance of 1.5 feet from the substrate and 3–7 kV of charge voltage gives better results for most types of substrate materials as compared to the current default settings used in the industry for standard electrostatic sprayers. It is safe to conclude that the electrostatic deposition of a nano-particle precursor disinfectant solution along with optimized system parameters characterized based on substrate type is very crucial for effective cleaning and disinfection. Gathering more such data can lead to the development of intelligent systems. The system developed in this study allows for nozzle changes with position feedback, without the need of manual intervention. This modification allows the system to be flexible and smart, making it efficient for use as a prospective autonomous and intelligent cleaning robot [92].

## Figures and Tables

**Table 1:** Sample preparation for SEM imaging.

Test Samples	Copper, Aluminum, Cast Iron, Stainless Steel, Acrylic, PVC, Polypropylene, Teflon, Rubber, Wood
Precursor Solution	Nanoxen
Sample Size	1${}^{\prime \prime }$ × 1${}^{\prime \prime }$
Nozzle Size	40 μm
Flow Rate	1.5 g/s
Voltage	ON|OFF
Ion Generator Output	7 kV
Working Distance	1.5 feet
Time of Spray	10 s

**Table 2:** Test matrix with varying voltage (V), nozzle spray size (ND) and working distance (WD).

Independent Variables	Case Matrix		
Spray Particle Size	40 µm, cone spray	80 µm, cone spray	110 µm, fan
Working Distance	0.5 feet	1.5 feet	2.5 feet
Charging Voltage	0 kV	3 ± 0.5 kV	7 ± 0.5 kV

## Data Availability

Not applicable.
